# KLF2 overrides the resident memory CD8 T cell differentiation program, in opposition to KLF3

**DOI:** 10.1073/pnas.2533700123

**Published:** 2026-03-23

**Authors:** Taylor A. DePauw, Kexin Gai, Jian Shen, Nicholas J. Maurice, Ka Hyun Rhee, William J. Valente, Christine H. O’Connor, Weiguo Cui, Changwei Peng, Stephen C. Jameson

**Affiliations:** ^a^Center for Immunology, University of Minnesota Medical School, Minneapolis, MN 55455; ^b^Department of Pathology, Feinberg School of Medicine, Northwestern University, Chicago, IL 60611; ^c^University of Minnesota Supercomputing Institute, Minneapolis, MN 55455

**Keywords:** memory CD8 T cells, KLF2, tissue-resident

## Abstract

Numerous transcriptional regulators have been associated with the differentiation pathways that lead to recirculating vs. tissue-resident memory T cells. However, it is unclear whether independent, coordinated expression of these regulators is required to determine residency vs. recirculation or whether there is a hierarchy, with some factors playing a dominant role in controlling T cell trafficking. We report that ablation of the gene encoding Kruppel-like factor 2 (KLF2) during CD8^+^ T cell activation leads to rapid transcriptional reprogramming, such that effector T cells fail to recirculate and prematurely acquire canonical phenotypic and transcriptional characteristics of resident memory cells (T_RM_). *Klf2*-deficient memory CD8^+^ T cells retained the capacity to undergo recall responses, including in vivo pathogen control. These data suggest that KLF2 diverts CD8^+^ T cells from the T_RM_ differentiation program. In contrast, ablation of another member of the KLF family, KLF3, enhanced differentiation of some recirculating T cell subsets and limited production of T_RM_ in lymphoid tissues. However, both KLF2 and KLF3 were required for differentiation of long-lived effector cells, suggesting cooperation between these factors in some situations. These findings indicate that KLFs occupy a central nexus in coordinating activated CD8^+^ T cell differentiation and trafficking.

## Significance

Memory T cells, generated after an immune response, fall into two main groups, depending on whether they circulate between tissues and blood or are “resident” in tissues. Various phenotypic and functional properties characterize recirculating and resident memory cells, but the transcription factors (TFs) that control differentiation of each group are unclear. We show that the TF Kruppel-like factor 2 (KLF2) regulates differentiation of recirculating memory T cells: Loss of KLF2 leads to generation of cells with characteristics of tissue-resident cells, which retained functionality. The related TF KLF3 opposes KLF2 in differentiation of some memory T cell subsets, while KLF2 and KLF3 cooperate for differentiation of others. These studies identify key transcriptional networks that control memory T cell trafficking

Following an immune response to an acute infection or immunization, memory CD8^+^ T cells are established. These can be divided into several subsets, including “central” and “effector” memory cells (T_CM_ and T_EM_, respectively), long-lived effector cells (LLEC), and tissue-resident memory cells (T_RM_) (1, 2). T_RM_ are distinguished from all other subsets by their lack of recirculation through blood and lymph. T_RM_ have been recognized as a key component in the recall immune response, mediating local protection in diverse tissues (3, 4). While sharing some features with T_EM_, T_RM_ show numerous transcriptional, epigenetic, and functional differences from their recirculating counterparts in mice and humans (3, 5, 6). Although there is variation between the transcriptional profiles and phenotype of T_RM_ found in different tissues, likely reflecting the impact of local cues, shared features of T_RM_ have been defined (7, 8). These include reduced expression of factors that mediate tissue egress and enhanced expression of factors normally associated with lymphocyte activation and/or exhaustion. T_RM_ are not solely found in nonlymphoid tissues, but are also detected in lymphoid sites, such as the spleen and lymph nodes (9), demonstrating coexistence of recirculating and resident cells in the same organs.

The basis for differentiation of CD8^+^ memory precursors into recirculating (T_CIRCM_) vs. resident subsets has been intensely investigated (3, 4, 6, 10–12). Several transcriptional regulators have been defined that distinguish T_RM_ from T_CIRCM_ and some have been proposed to promote generation of T_RM_, in at least some nonlymphoid tissues (NLTs) (including Runx3, Hobit, Blimp, Nr4a1, Ahr, Bhlhe40, and Notch/RBPJ), while others are suggested to support generation of T_CIRCM_ at the expense of T_RM_ (including KLF2, TCF-1, Zeb2, and Eomes) (10, 13). Dependency on a particular transcription factor (TF) may vary for T_RM_ in different tissue sites, and some TFs play a nuanced role: for example, strong expression of T-bet impairs generation of skin and lung T_RM_ but is required to generate T_RM_ in the liver, and low T-bet expression is required for maintenance of the skin T_RM_ pool (14–16). This also highlights the recognition that T_RM_ in distinct tissue sites have different characteristics and requirements for their generation (7, 16), although there has been progress in identifying properties of T_RM_ that are shared across all or most tissues (8). Several TFs promote or repress each other’s expression, suggesting a complex web of interconnected transcriptional regulation programs that determine subset generation (3, 10, 13). It is unclear, however, whether and which individual TFs play a dominant role in the differentiation of T_RM_ or T_CIRCM_, dictating the expression of other TFs and functional/phenotypic traits that distinguish these subsets.

In earlier studies, we showed that expression of the Kruppel-like factor KLF2 is extinguished during differentiation of CD8^+^ T_RM_ but maintained in T_CIRCM_ (17). Indeed, reduced expression of KLF2 is one of a few consistent “core” features that distinguish T_RM_ in varied tissues from T_CIRCM_, in mice and humans (3, 8, 12). Furthermore, we found that forced expression of KLF2 derails generation of T_RM_, consistent with loss of KLF2 being a critical step in establishment of T_RM_ (17). A well-defined target of KLF2 is sphingosine-1-phosphate receptor 1 (S1PR1), which has a well-defined role in lymphocyte egress from tissues (18, 19): Consistently, forced expression of S1PR1 could also oppose T_RM_ production, indicating that transcriptional control of *S1pr1* is relevant for the T_CIRCM_ vs. T_RM_ decision (17). It was therefore possible that the sole key role of KLF2 in regulating T_CIRCM_/T_RM_ generation was to promote S1PR1 expression, spoiling production of T_RM_ by permitting egress from tissues. Alternatively, KLF2’s role may extend beyond this activity, promoting the differentiation of T_CIRCM_ by coordinating expression of diverse genes including other TFs. Furthermore, recent studies indicate that KLF2 may act to preserve functional properties of memory CD8^+^ T cells, protecting cells from exhaustion in the context of chronic antigen exposure (20, 21), and in one report it was proposed that KLF2-deficient cells responding to acute infection were driven into a dysfunctional exhausted state (22).

Another member of the KLF family, KLF3, has also been suggested to influence differentiation and trafficking of lymphocytes. Studies in B cells indicate that KLF3 controls expression of β7 integrin and shapes follicular vs. marginal zone B cell differentiation (23–25). Like KLF2, KLF3 is decreased in expression in T_RM_, and it has been proposed, based on transcriptional network analysis, that KLF3 may cooperate with KLF2 in promoting T_CIRCM_ over T_RM_ differentiation in T cells (12). This has not been experimentally tested, however.

Since loss of KLF2 compromises thymic egress and naïve T cell homeostasis (26–28), we employed CRISPR/Cas9 approaches to ablate *Klf2* in mature CD8^+^ T cells, then assessed the impact on memory subset generation during an immune response in vivo. We show that loss of KLF2 leads to loss of recirculation into the blood and precocious differentiation of cells with the phenotype and gene expression profiles of T_RM_ in secondary lymphoid organs (SLO-T_RM_). Altered gene expression includes regulation of other transcription factors that have been associated with controlling T_CIRCM_/T_RM_ differentiation, including Hobit and Zeb2, as well as various egress factors (S1PR1, S1PR4, S1PR5). These data indicate that KLF2 plays a dominant role in restraining T_RM_ differentiation, such that loss of KLF2 leads to efficient and rapid T_RM_ induction. Nevertheless, in vitro and in vivo assays showed that *Klf2*-deficient memory CD8^+^ T cells retain functionality, including pathogen control in a recall response. Comparing genomic occupancy of KLF2 with loci that are differentially accessible in T_CIRCM_ vs. T_RM_ suggests KLF2 is largely associated with loci that are selectively expressed in T_CIRCM_, suggesting that KLF2 directs the T_CIRCM_ fate at the expense of T_RM_ differentiation. We also investigated the impact of KLF3 deletion. Interestingly, rather than paralleling the effects of KLF2 loss, deletion of *Klf3* preserved the capacity of effector and memory CD8^+^ T cells to recirculate and promoted differentiation of T_CM_-phenotype cells. Detailed comparison between the impact of *Klf2* and *Klf3* deletion suggests that these factors oppose each other in controlling differentiation of some T_CIRCM_ memory CD8^+^ T cell subsets, while cooperating in regulating differentiation of others. Together, these findings suggest KLF2 expression opposes resident memory CD8^+^ T cell differentiation, while KLF3 restrains KLF2 activity.

## Results

### Loss of KLF2 Expression Leads to Altered Tissue Distribution and Acquisition of T_RM_ Phenotype.

**Fig. 1.**
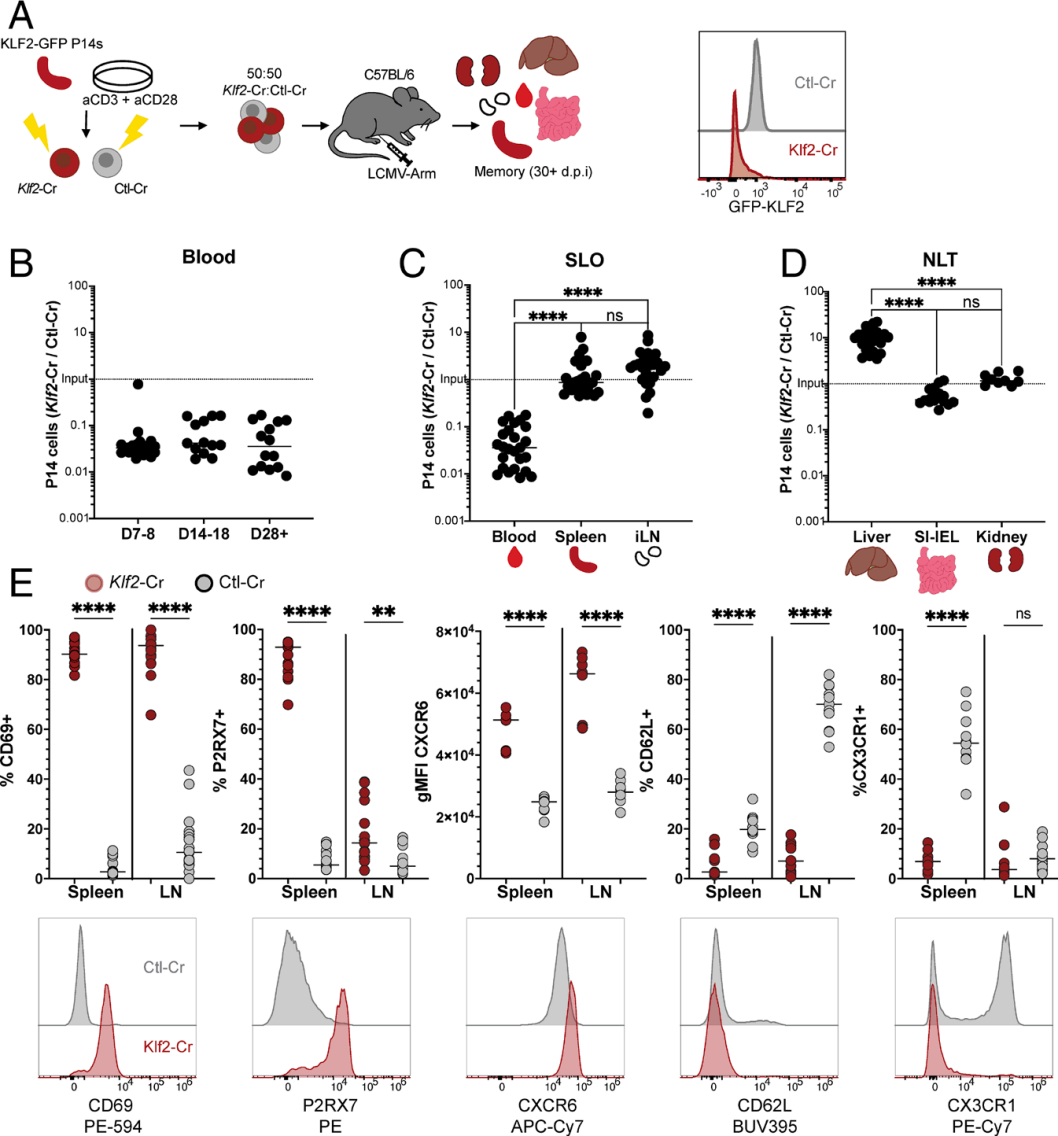
Loss of KLF2 in CD8^+^ T cells leads to alterations in trafficking and phenotype. (*A*) *Left*: experimental design schematic. P14 KLF2-GFP CD8^+^ T cells were subjected to CRISPR using sgRNAs for *Klf2* or a control gene (*Cd19* or *Thy1*) and equal numbers of congenically distinct cells were cotransferred into recipient mice, followed by LCMV-Arm infection. At the indicated times, donor cells were analyzed and/or isolated based on the expression of congenic markers (CD45 alleles). *Right*: representative histogram of KLF2-GFP expression in donor P14 cells subjected to CRISPR for *Klf2* vs. control, isolated from the spleen on d42 post–LCMV infection. (*B*–*E*) At least 28 d after infection (unless otherwise specified), cells were isolated from indicated tissues and relative frequencies of donor *Klf2*-Cr and control P14 KLF2-GFP T cells were determined. Data are compiled from 4 to 5 separate experiments. (*B*) shows the ratio among cells in blood at indicated time points, (*C* and *D*) shows the ratio among cells in lymphoid (*C*) and nonlymphoid tissues (*D*) at memory timepoints (D28+). (*E*) shows phenotypic analysis of Ctl-Cr (gray) and *Klf2*-Cr (red) P14 KLF2-GFP donor cells isolated from the spleen. Representative histograms are presented, together with compiled data from 3 to 5 experiments. Ordinary one-way ANOVA (*C* and *D*) and paired Student’s *t* test (*E*) were used for statistical analyses.

KLF2 expression is required for mature T cell egress from the thymus and for normal homeostasis of the peripheral naïve T cell pool (26–28). In order to interrogate the effects of KLF2 during the effector and memory stages of an immune response, we employed CRISPR/Cas9 approaches to ablate *Klf2* in in vitro activated P14 CD8^+^ T cells, then cotransferred those cells with congenically distinct P14 cells that were subjected to CRISPR for a control gene (*Thy1* or *Cd19*) into mice that subsequently received LCMV Armstrong infection (Fig. 1*A*). To monitor the efficacy of CRISPR, we used P14 cells carrying a KLF2 reporter (which encodes a GFP-KLF2 fusion protein). We confirmed efficient KLF2 deletion in splenic P14 cells that underwent CRISPR for *Klf2* (“*Klf2*-Cr” group) by loss of GFP-KLF2 expression compared to P14 cells subjected to CRISPR for the control gene (“Ctl-Cr” group) (Fig. 1*A* and *SI Appendix*, Fig. S1*A*), and by Sanger sequencing and ICE analysis (*SI Appendix*, Fig. S1*B*).

In naïve T cells, expression of KLF2 is essential for normal recirculation through lymphoid tissues, since KLF2 is required for expression of S1PR1 (needed for SLO egress) and CD62L (needed for access to most LNs) (26, 29). However, the role of KLF2 expression in controlling trafficking of effector CD8^+^ T cells is unclear. We observed that 7 to 8 d after initiating the response to LCMV, *Klf2-*Cr P14 CD8^+^ T cells were severely underrepresented in the blood, relative to Ctl-Cr P14 (Fig. 1*B*). This deficit in blood *Klf2*-Cr P14 cells extended through to memory time points (≥day 28) (Fig. 1*B*).

Such findings could indicate that KLF2 deficiency leads to death of the effector CD8^+^ T cells, and early studies on KLF2 suggested it was an important quiescence factor, preventing dysregulated proliferation and subsequent cell death (30, 31). However, examination of lymphoid tissues revealed that *Klf2*-Cr and Ctl-Cr P14 T cells were present at similar frequencies in SLOs (spleen and LNs) (Fig. 1*C*). We also examined the representation of *Klf2*-Cr cells in nonlymphoid tissues, finding that they there were substantially overrepresented in the liver but at similar frequencies as Ctl-Cr P14 T cells in the kidney and small intestine intraepithelial lymphocyte (SI-IEL) pools (Fig. 1*D*). Since CD8^+^ T cells in NLT are thought to derive from cells trafficking via the blood, a site in which *Klf2*-Cr cells are profoundly underrepresented (Fig. 1*B*), the finding that *Klf2*-Cr cells are present at similar (kidney, SI-IEL) or elevated frequencies (liver) suggests those cells have a competitive advantage over control populations in forming NLT memory populations. Together, these data indicate loss of KLF2 does not compromise survival of activated CD8^+^ T cells but does lead to profound redistribution of effector and memory CD8^+^ T cells, reducing the appearance of cells in the blood while maintaining or enhancing their abundance in tissues.

Since a central focus of these studies was whether loss of KLF2 would affect generation of memory CD8^+^ T cells with features of T_RM_, we analyzed *Klf2*-Cr and control populations for expression of canonical T_RM_ and T_CIRCM_ markers at 28 to 66 d post-LCMV. *Klf2*-Cr cells in the spleen and LNs showed uniform expression of CD69, while this marker only stained a small minority of control cells (Fig. 1*E*). While transient CD69 upregulation is associated with T cell activation, sustained CD69 expression by memory CD8^+^ T cells is widely used as a marker for T_RM_, often accurately predicting tissue residency when measured directly (e.g., via parabiosis) (8, 32). CXCR6 and P2RX7 expression have all been reported for T_RM_ in some tissue sites, and all were significantly elevated (in frequency and/or level of expression) in *Klf2*-Cr cells relative to the controls in SLOs (Fig. 1*E*). Some differences were observed for cells in spleen vs. LNs, however: e.g., P2RX7+ *Klf2*-Cr cells were abundant in spleen but rare in the LNs (Fig. 1*E*). On the other hand, CD62L and CX3CR1 expression is associated with recirculating memory CD8^+^ T cell populations (T_CM_ and subsets of T_EM_, respectively) and expression of these markers was reduced among the *Klf2*-Cr population (Fig. 1*E*). KLF2 is known to promote transcription of *Sell* (the gene encoding CD62L) (26, 29, 33), and these data indicate a role for KLF2 in controlling CX3CR1, CXCR6 and P2RX7 expression.

Similar phenotypic analysis was conducted in SI-IEL, kidney, and liver, as representative NLTs (*SI Appendix*, Fig. S1*A*). As expected, most Ctl-Cr P14 cells in the SI-IEL were low for KLF2-GFP expression and were CD69+, reflecting the known loss of KLF2 expression in SI-IEL T_RM_ (17) (*SI Appendix*, Fig. S1*A*). In the kidney and especially the liver, there were higher frequencies of CD69- KLF2-GFP+ cells among the Ctl-Cr group (*SI Appendix*, Fig. S1*C*), in keeping with our previous findings (17) and correlating with lower frequencies of resident memory cells in those sites (8, 32). In contrast, *Klf2*-Cr P14 in the kidney and liver were uniformly low for KLF2-GFP and CD69+ (*SI Appendix*, Fig. S1 *A* and *C*). Likewise, other phenotypic characteristics associated with CD8^+^ T_RM_, being P2RX7+, CXCR6+, CX3CR1−, and CD62L− was enhanced in the *Klf2*-Cr population of the kidney and, most markedly, the liver (*SI Appendix*, Fig. S1*A*). CD49a, which has also been used to identify T_RM_ in some tissues, was also elevated in *Klf2*-Cr P14 CD8^+^ T cells in both lymphoid and nonlymphoid tissues. As a technical note, we observed that it was critical to use a blocking nanobody against ARTC2.2 in order to detect the marker P2RX7 in the liver but not in others (spleen, LN, kidney, or SI-IEL) (*SI Appendix*, Fig. S1*A*), likely reflecting ARTC2.2-mediated cleavage of P2RX7 during cell isolation. Together, these data indicate that loss of KLF2 promotes the acquisition of phenotypic characteristics associated with T_RM_. CD103 (Integrin-αE) is strongly expressed by T_RM_ in some tissues, such as the SI-IEL—however, we did not see changes in CD103 expression by *Klf2*-Cr cells, relative to controls (*SI Appendix*, Fig. S1*D*).

### KLF2 Deficiency Leads to a T_RM_ Gene Expression Profile.

**Fig. 2.**
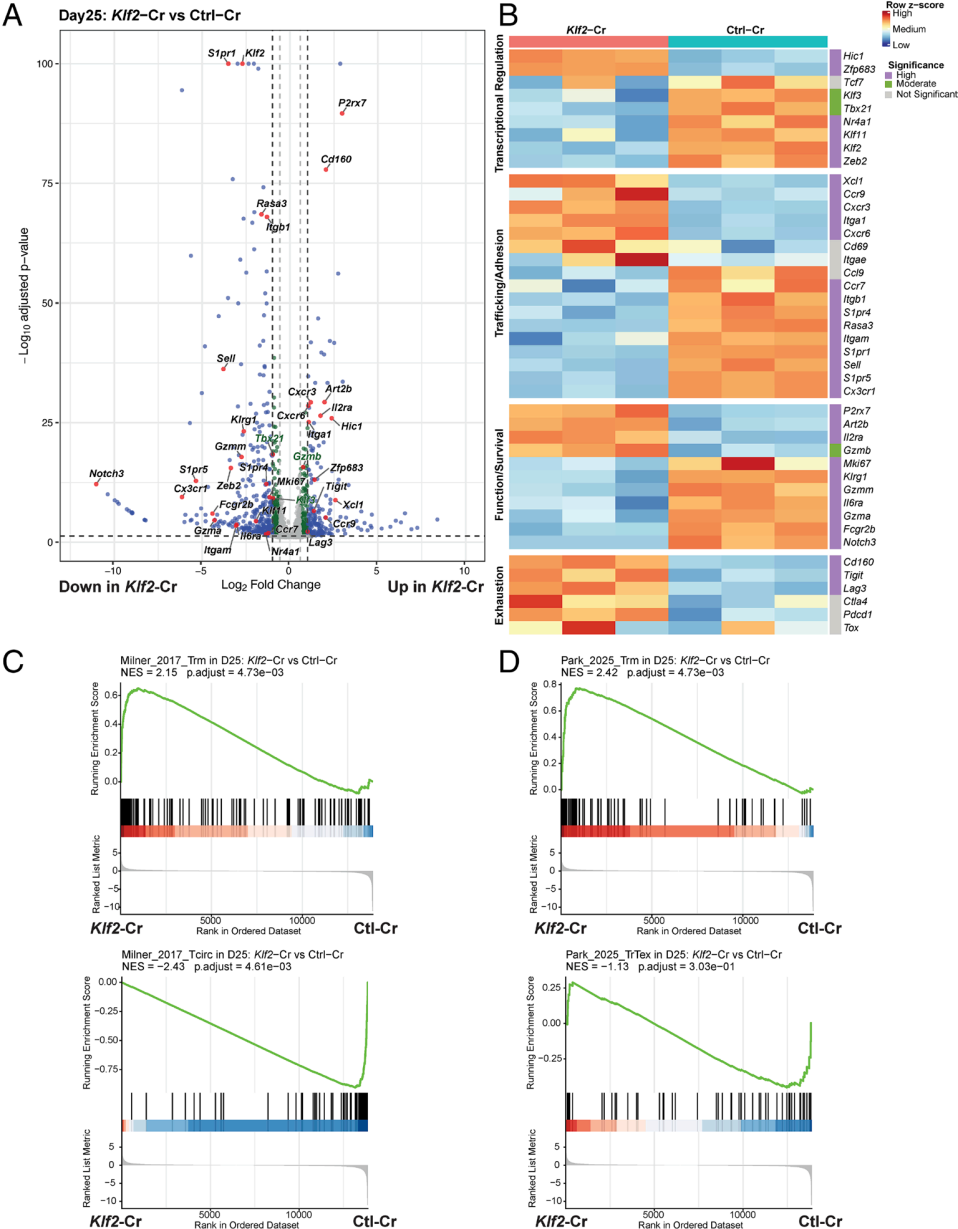
KLF2 deficiency results in a T_RM_-like gene expression profile. (*A*–*D*) Equal numbers of *Klf2*-Cr and control-treated P14 CD8^+^ T cells were cotransferred into WT recipients followed by LCMV infection as in Fig. 1. Both populations of donor cells were sorted, from three mice, on day 25 after LCMV infection and subjected to bulk RNA sequencing. (*A*) shows a volcano plot of differentially expressed genes in *Klf2*-Cr vs. control P14 cells. Genes significantly upregulated or downregulated (> 2-fold change) in *Klf2*-Cr cells are in blue and genes moderately differentially expressed (1.5 ~ 2-fold change) are in green. Genes of interest that are differentially expressed (*P* < 0.05) are labeled in red. All other genes that were not significantly changed are shown in gray. Cutoffs are indicated at log_2_FC = ±1 and *P*adj = 0.05 (gray lines). −log_10_(*P*adj) values were capped at 100 for visualization clarity. In (*B*), heatmaps for expression of genes involved in Transcription Regulation, Trafficking/Adhesion, Function/Survival, and Exhaustion are shown with relative expression across samples. Genes for which differential expression was significant (log_2_FC > 1, *P* < 0.05) are marked with purple, genes with moderate (1 ≥ log_2_FC ≥ 0.58, *P* < 0.05) differential expression are marked in green, and genes where significance thresholds were not met (*P* > 0.05) are in grey. (*C* and *D*) shows the result of GSEA, in which genes that were differentially expressed in the *Klf2*-Cr vs. Ctl-Cr RNAseq data was compared to the “Trm,” “Tcirc” gene signatures derived from Milner et al. (38) (*C*) and the “Trm” and “TrTex” signatures from Park et al. (39) (*D*).

Our phenotypic analysis included a limited set of cell surface markers that are frequently used to distinguish recirculating and resident CD8^+^ T cells. To explore the impact of *Klf2* deficiency more broadly, we examined the transcriptional profile of these cells. *Klf2*-Cr and cotransferred Ctl-Cr P14 splenocytes were isolated 25 d post–LCMV infection and subjected to RNA-seq. Fig. 2*A* shows a volcano plot of these data, identifying some differentially expressed genes between the control and *Klf2*-Cr groups (the complete gene expression analysis is provided in Dataset S1). Labeled genes include ones examined at a phenotypic level, including P2RX7, CXCR6, CD49a (encoded by *Itga1*) which echoed the cell surface protein expression characteristics reported in Fig. 1. KLF2 is known to promote S1PR1 gene expression (26, 29, 33) and consistently, *S1pr1* transcripts were markedly lower in the *Klf2*-Cr population (Fig. 2*A*). Counterintuitively, *Cd69* transcripts were only modestly (and not significantly) increased in the *Klf2*-Cr group, in contrast to the substantial elevation of cell surface CD69 expression observed in these cells (Fig. 1*E*). This can be explained because of the low expression of S1PR1 in *Klf2*-Cr cells: CD69 and S1PR1 are inversely correlated, due to interactions between these proteins that leads to their mutual destruction (34, 35). Because of this, transcription of *Cd69* in T cells is not observed as CD69 protein expression when S1PR1 levels are sufficient to target CD69 for degradation, but when S1PR1 expression is low, CD69 protein expression is detected. Thus, CD69 often serves as a marker of T cells with low S1PR1 expression (17, 36, 37). Our data suggested reduced S1PR1 expression (rather than elevated CD69 expression) accounts for the high cell surface CD69 protein levels on *Klf2*-Cr cells, consistent with our earlier studies (17, 36, 37). Flow cytometric detection of S1PR1 on ex vivo peripheral T cells is difficult (at least in part because of S1P-induced S1PR1 internalization), precluding direct testing of this hypothesis.

Some notable differences in gene expression between *Klf2*-Cr and control groups are highlighted in a heat map, organized by functional groups (Fig. 2*B*). Expression values are provided in *SI Appendix*, Fig. S2*A*. Aside from loss of *S1pr1* transcripts, *Klf2*-Cr cells showed increased expression of *Cxcr3*, *Cxcr6,* and *Ccr9*, and reduced *Sell* (encoding CD62L), *Cx3cr1*, *S1pr4,* and *S1pr5* mRNA (Fig. 2 *A* and *B*). This latter is relevant because studies have indicated that, in some tissues, S1PR5 may restrain induction of T_RM_, similar to S1PR1 (40). The result was surprising, however, because prior collaborative studies indicated that S1PR5 was under transcription control of Zeb2 (and, upstream of Zeb2, T-bet), in a pathway that runs parallel to KLF2 regulation of S1PR1 (40). However, consistent with that model, we also found a marked loss of *Zeb2* expression (and a more modest but significant decline in transcripts for *Tbx21*, the gene encoding T-bet), in *Klf2*-Cr cells (Fig. 2 *A* and *B*). Also of note, we observed reduced expression of *Rasa3* in *Klf2*-Cr cells. RASA3, a Rap1 and Ras GTPase-activating protein, has recently been shown to suppress LFA-1 activation, thereby playing an essential role in T cell tissue distribution, including lymph node entry and egress (41). These findings suggest distinct mechanisms through which KLF2 may regulate T cell trafficking. We also detected a small but significant increase in transcripts for *Hobit* (a gene also called *Zfp683* in mice and *Znf683* in humans) (Fig. 2 *A* and *B* and *SI Appendix*, Fig. 2*A*). Hobit has been shown to promote generation of T_RM_ in some tissues, and it has been suggested that Hobit may repress KLF2 and some of its target genes (10, 42). Elevated *Hobit* expression in *Klf2*-Cr cells suggests the opposite may also occur, with *Hobit* expression being elevated by KLF2 deficiency. Expression of *Hic1*, which has been associated with generation of SI-IEL T_RM_ (43), was also increased in *Klf2*-Cr CD8^+^ T cells. In addition to P2RX7, *Klf2*-Cr cells showed altered expression of other molecules associated with function, activation, and survival, including increased gene expression of *Il2ra* and reduced expression of *Klrg1*, *Gzma,* and *Il6ra* (Fig. 2 *A* and *B*). It is worth noting the *Mki67* was reduced in expression in *Klf2*-Cr cells, which is inconsistent with the proposal that KLF2 acts as brake on proliferation (Fig. 2 *A* and *B*) (30, 31). Interestingly, we also observed that several genes typically associated with CD8^+^ T cell exhaustion were increased in *Klf2*-Cr cells: this included elevated *Lag3*, *Tigit,* and *Cd160*, and a trend to increased *Pdcd1* (encoding PD-1), *Ctla4* and *Tox* (Fig. 2*E*).

We next used gene set enrichment analysis (GSEA) to assess whether the transcriptional differences in *Klf2*-Cr cells align with reported T_RM_ and T_CIRCM_ gene expression signatures. Comparison with two datasets (8, 38) indicated a strong correlation between genes that define a consensus T_RM_ signature with the differentially expressed genes of the *Klf2*-Cr group, and a negative correlation with the T_CIRCM_ gene expression signature (Fig. 2 *C* and *D* and *SI Appendix*, Fig. S2*B*). This includes but is not limited to many of the specific genes discussed above. One of the datasets chosen for alignment consists of a recently defined “Core T_RM_ signature,” comprising genes that show elevated or diminished expression in T_RM_ from numerous tissues and infection conditions, compared to recirculating memory T cell populations (8) (*SI Appendix*, Fig. S2*B*).

As noted, several markers associated with T cell exhaustion were induced in KLF2-deficient CD8^+^ T cells (Fig. 2 *A* and *B*). Some of these factors were previously reported to be increased in T_RM_ (43), suggesting that they may be shared features of resident and exhausted CD8^+^ T cells. Very recent studies have identified gene expression signatures that distinguish T_RM_ produced by acute infection from a tissue-resident population of exhausted T cells (Tr-T_EX_), produced during chronic infection (39). GSEA showed that *Klf2*-Cr cells had a significant positive correlation with the T_RM_ gene expression signature, while there was a negative correlation (albeit nonsignificant) with Tr-T_EX_ (Fig. 2*E*). This analysis reinforces the interpretation that KLF2 deficiency leads to generation of cells with the transcriptional profile of T_RM_ following acute infection.

We investigated whether KLF2 loss promoted acquisition of this T_RM_-like gene expression early in the CD8^+^ T cell immune response. *Klf2*-Cr and Ctl-Cr P14 cells were isolated at day 7 following the response to LCMV Armstrong and subjected to RNAseq (*SI Appendix*, Fig. S2 *D*–*F*) (Dataset S2). This is close to the peak of the effector response to LCMV, yet even by this timepoint, most gene expression differences that we observed at day 25 were already observed. Furthermore, GSEA of these data suggested that the changes in gene expression aligned with acquisition of a T_RM_-like transcriptome by effector-phase *Klf2*-Cr cells (*SI Appendix*, Fig. S2 *E* and *F*).

Hence, by transcriptional analysis, *Klf2*-Cr CD8^+^ T cells in the spleen acquire many characteristics of T_RM_, and this is observed at both effector and memory time points, suggesting that *Klf2* loss results in rapid deflection from the normal differentiation trajectory of activated CD8^+^ T cells and leads to precocious acquisition of a resident memory-like state.

### KLF2-Deficient Memory CD8^+^ T Cells Retain Functionality.

While our GSEA studies suggested *Klf2*-Cr cells had the gene expression signature of T_RM_ rather than their exhausted counterparts (39) (Fig. 2*E*), a recent report by Fagerberg et al. argued that KLF2-deficient memory P14 CD8^+^ T cells, produced in response to acute LCMV infection, showed features of exhaustion and were defective in recall expansion after adoptive transfer and stimulation (22). Hence, we further explored the functionality of *Klf2*-Cr memory CD8^+^ T cells, in terms of their in vitro and in vivo responses and ability to control pathogens.

**Fig. 3.**
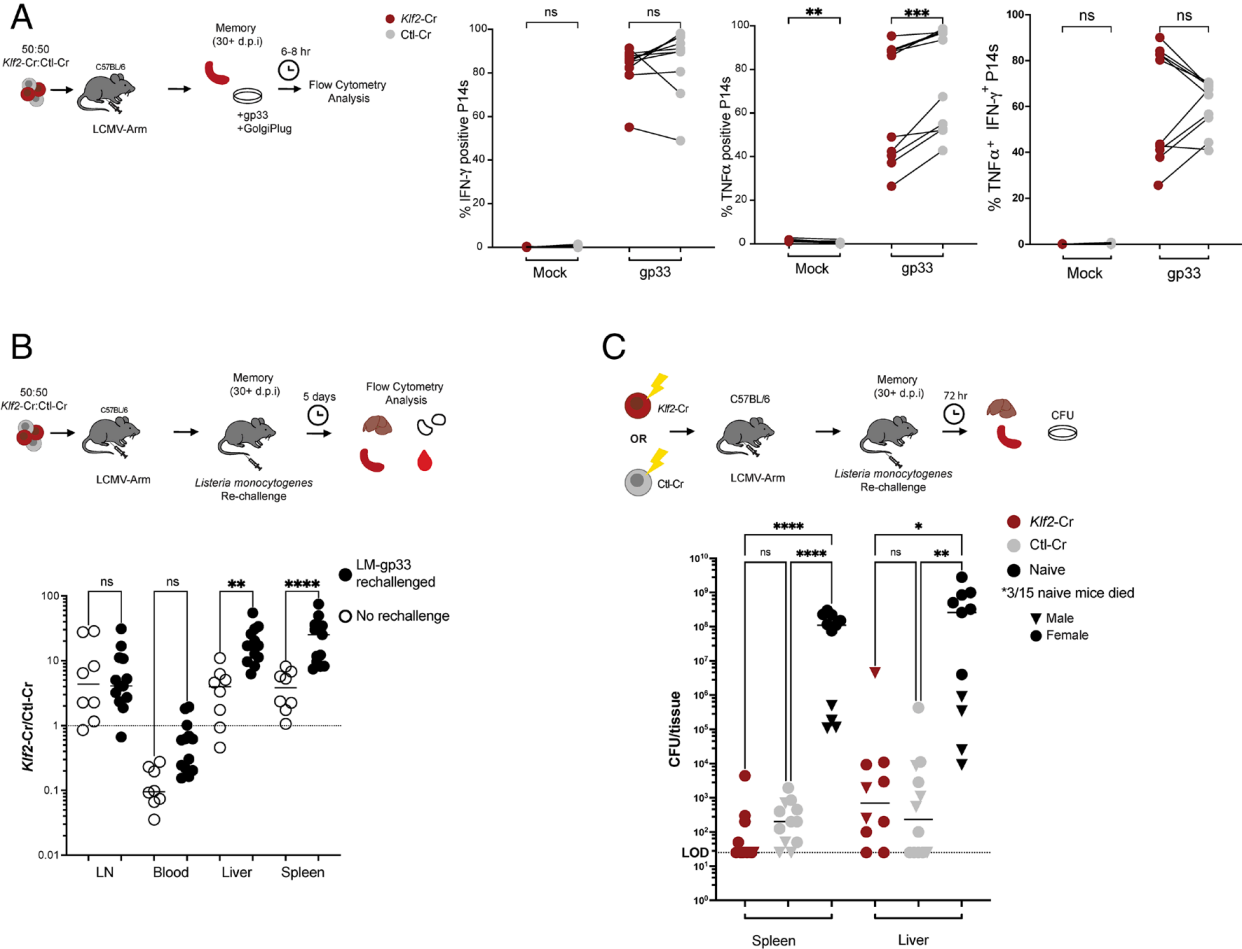
KLF2 deficiency memory CD8^+^ T cells retain functionality. Congenically distinct *Klf2*-Cr and *Thy1*-Cr P14 cells were cotransferred (*A* and *B*) or individually transferred (*C*) into C57BL/6 recipients, which were then infected with LCMV Armstrong and maintained for at least 30 d. (*A*) Splenocytes were isolated at least 28 d post–LCMV-Armstong infection and cultured with gp33 peptide for 4 to 6 h in the presence of GolgiPlug, then donor P14 populations were analyzed for intracellular IFN-γ and TNF. (*B*) P14 chimeras were infected with Lm-gp33 or left unchallenged. Five days later, the mice were euthanized, and the ratio of *Klf2*- and *Thy1*-Cr P14 cells was determined in the indicated tissues. In (*C*), animals receiving either *Klf2*- or *Thy1*-Cr P14 cells were infected with high-dose Lm-gp33 and CFU determined on day 3. C57BL/6 mice receiving neither P14 cells nor LCMV were used as a naïve control. Paired (*A*) and Unpaired (*B* and *C*) Student’s *t* test was used for statistical analyses.

For initial characterization, *Klf2*-Cr (and control *Thy1*-Cr) P14 memory CD8^+^ T cells were stimulated in vitro with gp33 peptide and assayed for cytokine production and proliferation. We observed a significant difference in the frequency of *Klf2*-Cr cells producing TNF, reflecting an average reduction of 1.25 (±0.23) fold, but we found no significant difference in the ability of *Klf2*-Cr cells to produce IFN-γ or to coproduce IFN-γ and TNF, and no significant change in the expression levels of each cytokine (Fig. 3*A* and *SI Appendix*, Fig. S3*A*). Consistently, Fagerberg et al. reported that *Klf2*-Cr memory cells retained the ability to produce TNF and IFN-γ (22). We also found that proliferation following in vitro activation was similar for *Klf2*- and *Thy1*-Cr memory P14 CD8^+^ T cells (*SI Appendix*, Fig. S3*B*).

Since in vivo assays provide a more physiologically meaningful readout of functionality, we next compared the recall response of coadoptively transferred *Klf2*- and *Thy1*-Cr memory P14 cells in mice infected with recombinant *Listeria* monocytogenes expressing the LCMV gp33 epitope (Lm-gp33), 5 d postchallenge (Fig. 3*B*). Control mice were not infected with Lm-gp33 to establish a basal ratio of the donor populations at memory. The ratio of *Klf2*- to *Thy1*-Cr cells was maintained or increased in the Lm-gp33 challenged mice, indicating that the *Klf2*-deficient population were fully competent to undergo recall proliferation (Fig. 3*B*). Indeed, in the target tissues of Lm-gp33 infection (spleen and liver) the expansion of the *Klf2*-Cr population evidently exceeded that of *Thy1*-Cr control cells in the same tissue (indicated by elevated ratios of *Klf2*-Cr to *Thy1*-Cr cells, relative to mice that did not receive Lm-gp33 infection), suggesting the *Klf2*-Cr population was superior in their proliferative response to Lm-gp33 challenge. Finally, we determined whether *Klf2*-Cr memory CD8^+^ T cells were competent to mediate pathogen control. *Klf2*-Cr and *Thy1*-Cr P14 cells were transferred separately into mice which were primed with LCMV Armstrong and at a memory timepoint the animals were challenged with Lm-gp33, and Lm control was measured 3 d later (Fig. 3*C*). Naïve mice, used as controls, showed high *Listeria* counts in the spleen and liver but mice that harbored either *Klf2*-Cr or *Thy1*-Cr P14 cells showed significant (and similar) reduction in Lm-gp33 bacterial load (Fig. 3*C*). These data demonstrate that, despite radical changes in their transcriptional profile and trafficking, the fundamental function of protective immunity was preserved in *Klf2*-deficient memory CD8^+^ T cells. Taken together, our data argue that *Klf2*-deficient cells do not display functional defects associated with T cell exhaustion, at least for the responses studied. Potential reasons for the contrasting conclusions of Fagerberg et al. are addressed in the discussion.

### KLF2 Binds to Candidate Regulatory Sites of Many Genes That Are Affected by KLF2 Deficiency.

Aside from a small group of known target genes, the genetic loci bound by KLF2 in T cells are largely uncharacterized. There is considerable redundancy in the consensus DNA binding sites of KLFs, making prediction difficult. Furthermore, since *Klf2*-Cr CD8^+^ T cells showed changes in numerous other transcription factors, it was possible that many of the differentially expressed genes are not direct targets of KLF2 regulation.

**Fig. 4.**
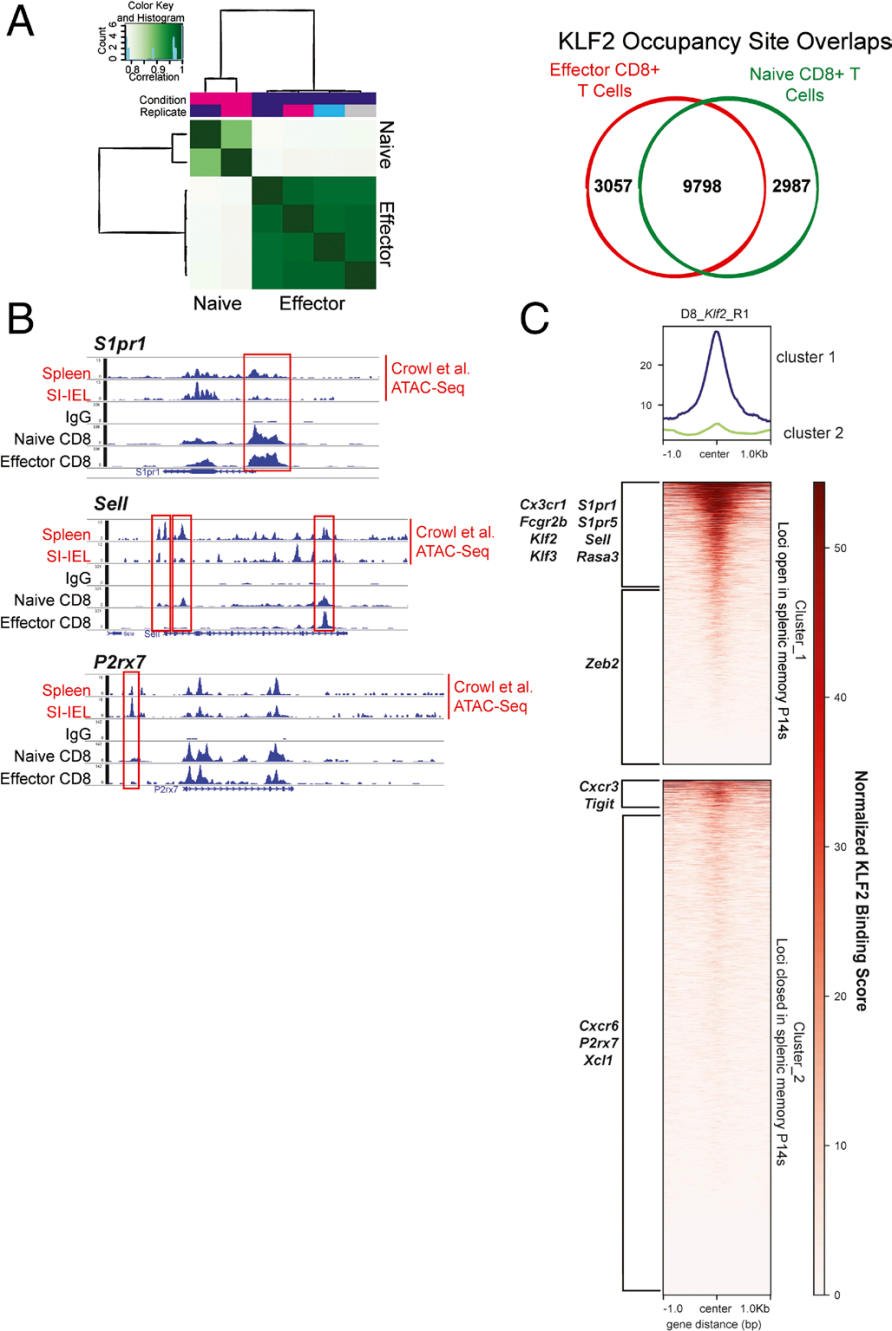
Genomic occupancy of KLF2 is primarily associated with loci that are accessible in recirculating memory CD8^+^ T cells. (*A*–*C*) KLF2 occupancy across the genome was determined by KLF2 CUT&Tag-seq of CD8^+^ T cells. CD8^+^ T cells were isolated from an unimmunized mouse (“Naïve”) or isolated from mice that had been infected with LCMV-Arm 8 d previously (“Effector”). Two naïve and two effector stage samples (the latter including a technical repeat) were analyzed, using antibody to KLF2 or an IgG control for CUT&Tag. (*A*) shows the similarity in KLF2 occupancy sites in naïve and effector CD8^+^ T cell samples (*Left*); and the average number of unique and overlapping KLF2-bound sites in naïve and effector CD8^+^ T cell samples (*Right*). (*B*) shows representative KLF2 occupancy at loci that include the *S1pr1*, *Sell*, and *P2rx7* genes, in naïve and effector CD8^+^ T cells. An IgG control is shown for comparison. (*C*) ATAC-seq loci that were differentially accessible in T_CIRCM_ and T_RM_ (*SI Appendix*, Fig. S4*B*) (43) were aligned with loci showing KLF2 occupancy from CUT&Tag analysis of effector CD8^+^ T cells. Overlapping regions were scored and normalized, to provide a ranked list of KLF2 binding at ATAC-seq DARs, displayed as a tornado plot. “Cluster 1” represents DARs that are more accessible in T_CIRCM_, while “Cluster 2” DARs are less accessible in T_CIRCM_ compared to T_RM_ populations (*SI Appendix*, Fig. S4*B*) (43). Sites where there was substantial KLF2 occupancy are indicated at the top of each cluster. The loci associated with some differentially expressed genes (Fig. 2*B*) are also highlighted in red boxes, alongside with ATAC-seq tracks from Crowl et al. showing DARs from spleen T_CIRCM_ and SI-IEL T_RM_ cells.

To begin addressing this, we examined KLF2 occupancy in CD8^+^ T cells using CUT&Tag-seq (Cleavage under target and TAGmentation) (Fig. 4*A*) (Dataset S3). CD8^+^ T cells were isolated from unimmunized mice and from mice infected with LCMV Armstrong 8 d earlier, in order to compare KLF2 occupancy in naïve and effector CD8^+^ T cells, respectively. KLF2 is constitutively expressed in naïve T cells and, while KLF2 is lost following T cell activation, expression is restored by day 5 of the response to LCMV-Arm (17, 44).

KLF2 occupancy was detected at many thousands of loci in naïve and d8 activated CD8^+^ T cells, with many of these sites overlapping between the time points (Fig. 4*A*) (Dataset S3). Profiles for KLF2 occupancy at selected gene loci are indicated in Fig. 4*B*, which also shows ATAC-seq peaks that were previously characterized in recirculating (spleen) and resident (SI-IEL) P14 memory cells (43). KLF2 is known to promote expression of *S1pr1* and *Sell*—both are expressed in naïve cells, while d8 effector cells express *S1pr1* but not *Sell*. KLF2 was associated with the putative promoter and enhancer sites (as well as other loci) in both genes in naïve cells, but one of the sites at the proximal enhancer for *Sell* was completely lost after activation. Notably, for both loci, KLF2 was found to occupy sites that are more accessible in T_CIRCM_ (spleen) than T_RM_ (SI-IEL), consistent with KLF2 playing a positive role in expression of *S1pr1* and *Sell*. On the other hand, KLF2 occupancy was also found adjacent to genes that were upregulated following *Klf2*-Cr, including *P2rx7*, which is strongly expressed by T_RM_ but is minimally expressed in naïve or T_CIRCM_ (45–47), perhaps indicating that KLF2 can also restrain gene expression (Fig. 4*B*). However, while KLF2 occupancy was detected at multiple sites in the *P2rx7* locus, there was minimal evidence of KLF2 binding at the peak that was preferentially open in T_RM_ relative to T_CIRCM_. KLF2 was found to occupy potential regulatory sites for many genes that were differentially expressed in our RNAseq studies, including genes that were increased or decreased in expression in CD8^+^ T_RM_. Examples of these are provided in *SI Appendix*, Fig. S4*A*. Hence, the significance of KLF2 in regulating gene expression was difficult to resolve by its occupancy alone.

To explore this issue further, we aligned the genomic loci occupied by KLF2 in d8 effector cells to sites that were found to be differentially accessible regions (DARs) in T_CIRCM_ and T_RM_, from ATAC-seq studies (43). To do this, we first compiled the published ATAC-seq data (43) into two clusters, one consisting of DARs that are all more accessible in splenic memory T cells (T_CIRCM_) compared to T_RM_ populations (Cluster 1), while loci were less accessible in splenic memory cells compared to T_RM_ were assigned to Cluster 2 (Dataset S4). A heat map showing the characteristics of these DARs, mapped onto the data from Crowl et al. is provided in *SI Appendix*, Fig. S4*B*.

Next, we used bioinformatic approaches to search for sites in which KLF2 occupancy overlaps with the DARs that distinguish T_CIRCM_ and T_RM_. The results are summarized in a tornado plot (Fig. 4*C*), which shows the DARs ranked by the normalized score of their overlap with KLF2 occupancy sites, and illustrates the position of KLF2 occupancy relative to the center of the ATACseq DARs. The tornado plot corresponds to the data provided in Dataset S5. In essence, this figure illustrates sites in which KLF2 occupancy correlates with DARs that are more (Cluster 1) or less (Cluster 2) accessible in T_CIRCM_ compared to T_RM_. Overall, we found that KLF2 strongly associated with DARs in Cluster 1, with much less evidence for KLF2 occupancy at DARs which are more accessible in Cluster 2, suggesting that KLF2 more frequently binds sites that are “opened” rather than “closed” in T_CIRCM_ (Fig. 4*C*). Based on the intensity of the normalized KLF2 occupancy score, we estimated that roughly 33% of Cluster 1 DARs matched with KLF2 binding, while this was true for only approximately 5% of Cluster 2 DARs.

We then wanted to determine how genes of interest that are differentially expressed in *Klf2*-Cr memory CD8^+^ T cells (Fig. 2*B*) correlate with DARs that exhibit overlapping KLF2 occupancy. A given gene locus may have multiple associated DARs, so we apply a criterion of there being at least one DAR which showed substantial KLF2 occupancy. With this threshold, we found that loci for several genes, including *Cx3cr1*, *Fcgr2b*, *Klf2, Klf3*, *S1pr1*, *S1pr5*, *Sell,* and *Rasa3,* had DARs that were associated with KLF2 occupancy in Cluster 1 (annotations in Fig. 4*C*). Expression of these genes was substantially reduced in KLF2-deficient memory CD8^+^ T cells (Fig. 2*B*). In contrast, in Cluster 2, only DARs associated with *Cxcr3* and *Tigit* (both of which show elevated transcription in *Klf2*-Cr cells) were found to be associated with KLF2 occupancy, albeit weakly. Together, these data suggest that KLF2 occupancy is primarily associated with DARs that are preferentially opened in T_CIRCM_ and with genes that appear to depend on KLF2 for their expression, arguing that KLF2 is typically acting to drive rather than repress transcription in CD8^+^ T cells. DARs associated with several other differentially expressed genes (Fig. 2*B*) showed minimal evidence for associated KLF2 binding, however. This includes *P2rx7*, *Cxcr6*, *Ccr9,* and *Xcl1* in Cluster 2, and *Zeb2* in Cluster 1 (Fig. 4*C*). Expression of these genes is presumably controlled by other transcription factors that are themselves regulated by KLF2. Indeed, previous work has shown that *Zeb2* is under the control of T-bet in CD8^+^ memory T cells (40), and that KLF2 promotes the transcriptional activity of T-bet (22), suggesting a potential regulatory circuit for *Zeb2* expression.

Hence, while we and others have proposed that KLF2 can both enhance and repress transcriptional activity in lymphocytes (33, 37, 48, 49), these findings suggest KLF2 occupancy more often correlates with transcriptional activation of loci expressed by T_CIRCM_, rather than repression of loci that are characteristically expressed in T_RM_.

### KLF3 Deficiency Limits T_RM_ Differentiation and Alters Representation of T_CIRCM_ Subsets.

As noted, expression of *Klf3* was reduced in *Klf2*-Cr cells, at both d25 and d7 post–LCMV infection (Fig. 2 *A* and *B*, *SI Appendix*, Fig. S2 *D* and *E*, and Datasets S1 and S2). KLF3 often acts as a repressor of gene expression (50, 51) and is known to cooperate with or antagonize other KLFs in various cell types (50–52). In B cells, KLF2 and KLF3 have been reported to have reciprocal effects on marginal zone (MZ) B cell differentiation, but both appear to be required for B1 B cell differentiation (23, 48, 53, 54). The role of KLF3 in memory T cell differentiation and regulation of trafficking is unclear—but *Klf3* expression is reduced in at least some T_RM_ subsets and it was suggested that KLF3 could cooperate with KLF2 in opposing generation of T_RM_ (12).

**Fig. 5.**
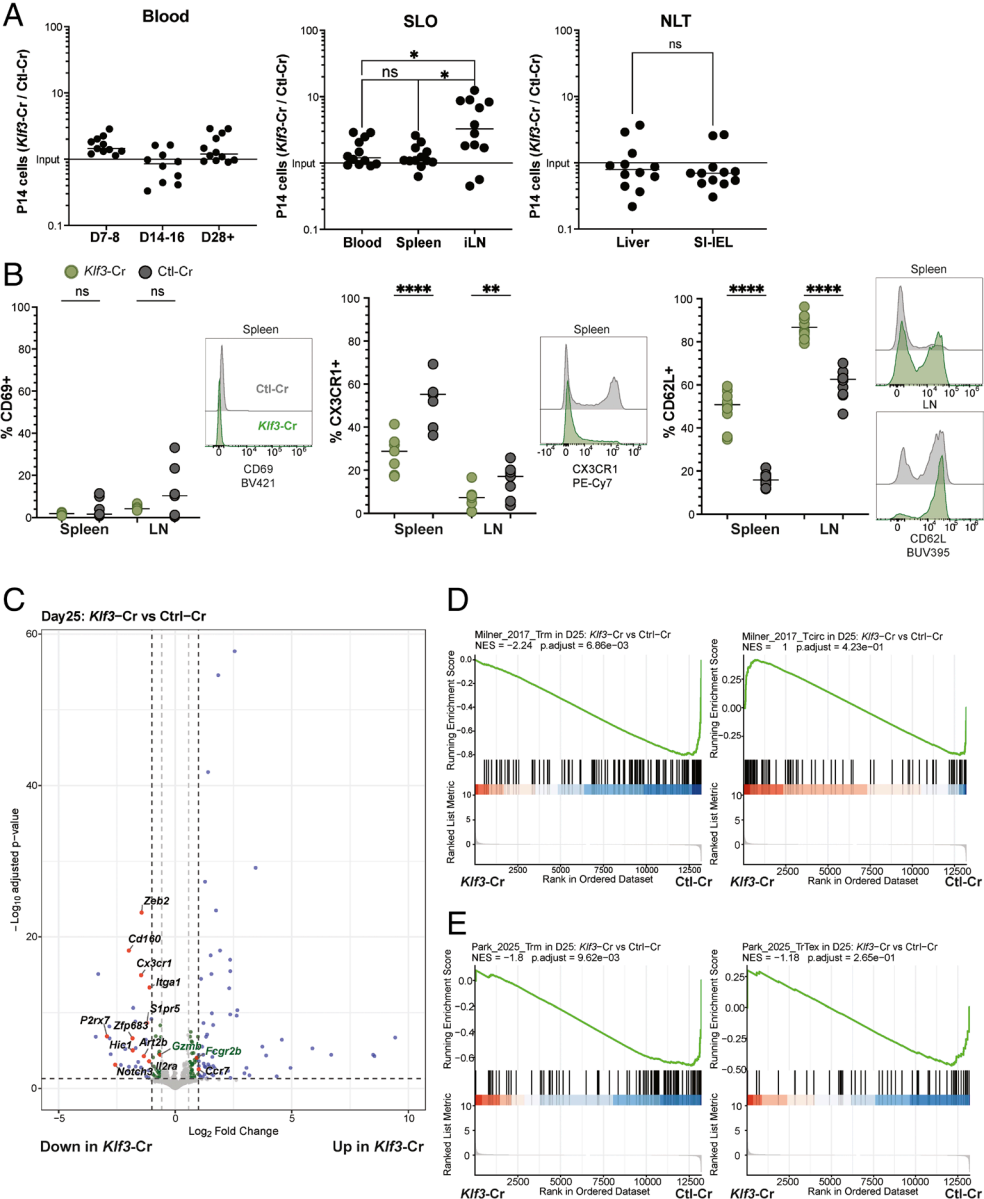
Loss of KLF3 tunes CD8^+^ T cell differentiation. (*A*–*E*) Equal numbers of congenically distinct *Klf3*-Cr and Ctl-Cr P14 CD8^+^ T cells were cotransferred into WT recipient mice, followed by LCMV-Arm infection, as in Fig. 1. (*A*) the ratio of donor P14 populations was measured in indicated tissue sites (at Day 28+ post-LCMV, unless noted), and (*B*) cells from indicated tissues were subjected to phenotypic analysis. (*C*–*E*) Donor *Klf3*- or Ctl-Cr cells were sorted at day 25 post–LCMV infection for RNA sequencing. (*C*) Shows a volcano plot of differentially expressed genes (using the same parameters as Fig. 2*A*), and (*D*–*E*) shows GSEA of these data (as described for Fig. 2 *C*–*D*). Ordinary one-way ANOVA (*A*, SLO) and paired Student’s *t* test (*A*, NLT, and *B*) was used for statistical analyses.

To investigate this, we used CRISPR/Cas9 to ablate *Klf3* in P14 GFP-KLF2 cells, using the same approaches as for *Klf2* (Fig. 1*A*). *Klf3* ablation was determined by ICE analysis (*SI Appendix*, Fig. S5*A*). Notably, we did not observe a reduction in the ability of *Klf3*-Cr cells to access the blood, at any time point (Fig. 5*A*)—indeed, there was trend toward modestly increased representation of *Klf3*-Cr cells in the blood. Similar to *Klf2*-Cr cells, *Klf3*-Cr cells have equal representation to control cells in the spleen but accumulated slightly in the lymph nodes (Fig. 5*A*). Regarding NLT, *Klf3*-Cr cells were underrepresented in SI-IEL, although to a lesser extent than *Klf2*-Cr cells, and also underrepresented in the liver (Fig. 5*A*), in contrast to *Klf2*-Cr cells which were overrepresented in the liver (Fig. 1*D*). The effects of *Klf3*-Cr were generally more muted in SLOs and NLTs compared to the impact of *Klf2*-Cr (Fig. 1 *C* and *D*), which might suggest a minimal impact of KLF3 loss. However, we considered that KLF3 may influence the representation of subpopulations of memory CD8^+^ T cells which might minimize the apparent impact of *Klf3* loss.

Phenotypic analysis indicated that KLF3 deficiency did not promote SLO-T_RM_ generation (Fig. 5*B* and *SI Appendix*, Fig. S5*B*). In fact, the rare CD69+ cells observed among control P14s in spleen and lymph nodes were almost completely absent in the *Klf3*-Cr population (Fig. 5*B*). Instead, we noted a slightly elevated representation of T_CM_ phenotype cells (i.e., CD69−, CX3CR1−, CD62L+) among the *Klf3*-Cr group (Fig. 5*B*).

RNAseq analysis of *Klf3*-Cr vs. Ctl-Cr cells performed at d25 of the response to LCMV supported these conclusions (Fig. 5*C*, *SI Appendix*, Fig. S5*C*, and Dataset S6). *Klf3*-Cr memory CD8^+^ T cells isolated from the spleen showed a reduction in prominent T_RM_ signature genes (*P2rx7*, *Itga1*, *Zfp683*) (Fig. 5*C* and *SI Appendix*, Fig. S5*C*). Given that we obtained very few SLO-T_RM_ in the control group (Fig. 5*B*), these changes may underestimate the impact of KLF3 loss. We also observed a trend toward increased expression of *Sell* and *S1pr1* (Fig. 5*C* and *SI Appendix*, Fig. S5*C*). On the other hand, and in keeping with the phenotypic data, there were some changes in gene expression in *Klf3*-Cr cells that echoed the effects of *Klf2* loss, including decreased expression of *Zeb2*, *Cx3cr1,* and *S1pr5*, in both groups (Fig. 5*C* and *SI Appendix*, Fig. S5*C*). Of the genes that were increased in expression in *Klf3*-Cr cells, few had defined roles in T cells (Fig. 5*C* and Dataset S6). Of note, *Klf3* transcripts were increased in *Klf3*-Cr cells [in keeping with data from studies in B cells which reported that KLF3 negatively regulates its own transcription (23)], while *Klf2* expression was not substantially changed.

We used GSEA to assess whether the transcriptional differences in *Klf3*-Cr cells aligns with reported T_RM_ and T_CIRCM_ gene expression signatures (Fig. 5*D* and *E* and *SI Appendix*, Fig. S5 *C* and *D*). As indicated from our phenotypic data, *Klf3*-Cr cells showed a significant negative correlation with a T_RM_ gene expression signature (but no significant correlation with the Tr-T_EX_ gene signature). However, when compared to a T_CIRCM_ gene expression signature *Klf3*-Cr cells had an overall positive correlation, but this was not significant. We considered that this might arise because of heterogeneity within the circulating pool of memory CD8^+^ T cells, if *Klf3*-loss differentially affected distinct subsets of T_CIRCM_.

### Opposing and Cooperative Regulation of CD8^+^ T Cell Differentiation by KLF2 and KLF3.

**Fig. 6.**
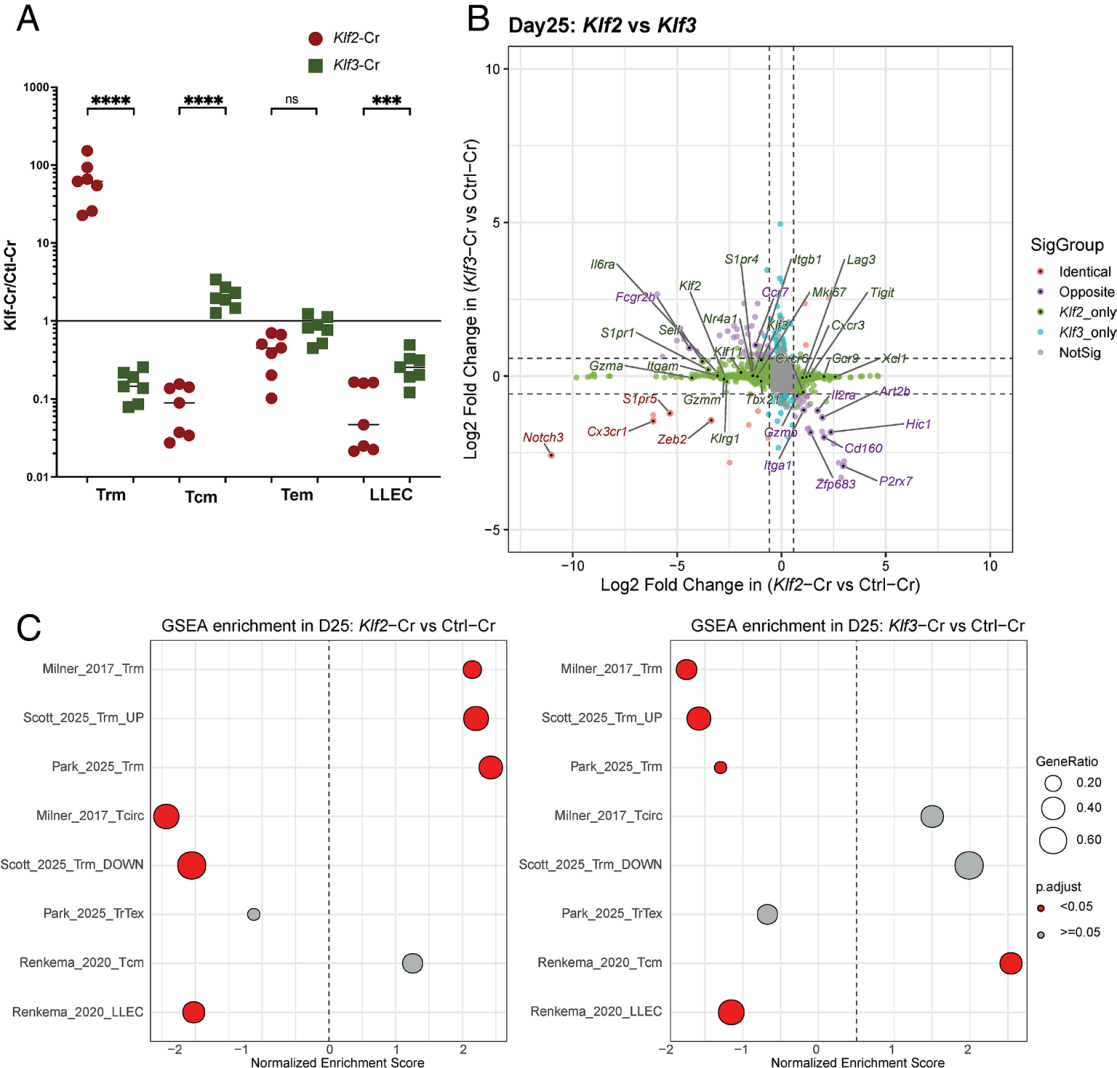
KLF2 and KLF3 deficiency leads to reciprocal and concordant changes in memory CD8^+^ T cell subset differentiation. (*A*) Shows the ratio of *Klf2*- and *Klf3*-Cr cells (relative to paired controls) among distinct subsets of circulating memory CD8+ T cells, and TRM. Subsets were defined as T_CM_ (CD62L+, CD69−, CX3CR1−), T_EM_ (CD62L−, CD69−, CX3CR1−), LLEC (CD62L−, CD69−, KLRG1+, CX3CR1+), and T_RM_ (CD69+, CD62L−, CX3CR1−) in samples isolated from the spleen at day 29 to 40 post–LCMV infection. (*B*) Gene–gene correlation plot for *Klf2*-Cr and *Klf3*-Cr RNA sequencing. (*C*) Dot plot graph showing GSEA results for all *Klf2*-Cr and *Klf3*-Cr GSEA comparisons. Ordinary one-way ANOVA was used for statistical analysis of (*A*).

To explore the idea that KLF3 deficiency may selectively affect certain circulating memory populations, we resolved splenic T_CIRCM_ into three major subsets composed of T_CM_, LLEC, and circulating T_EM_ (1). We used phenotypic analysis to distinguish T_CM_ (CD62L+, CD69−, CX3CR1−, KLRG1−), T_EM_ (CD62L−, CD69−, CX3CR1−, KLRG1−), and LLEC (CD62L−, CD69−, CX3CR1+, KLRG1+) and applied these to our studies on both *Klf2*-Cr and *Klf3*-Cr memory CD8^+^ T cells (gating strategy in *SI Appendix*, Fig. S6*A*). This analysis indicated reciprocal effects of *Klf2*-Cr and *Klf3*-Cr on the representation of T_CM_ and T_RM_ in the spleen: *Klf2*-targeting caused a substantial increase in the frequency of T_RM_-like cells and reduced representation of T_CM_ phenotype cells, while *Klf3*-Cr cells showed the opposite pattern (although the impact of *Klf3* deficiency was less extreme) (Fig. 6*A*). However, ablation of either *Klf2* or *Klf3* led to a reduced frequency of T_LLEC_ (Fig. 6*A*).

Likewise, reanalysis of the differential gene expression in *Klf2*-Cr and *Klf3*-Cr memory CD8^+^ T cells indicated concordant and discordant patterns of regulation (Fig. 6*B*). Correlating gene expression changes revealed that genes associated with T_RM_ (*P2rx7*, *Hic1*, *Zfp683*, *Art2b*) were increased in expression in *Klf2*-Cr populations but reduced in expression in *Klf3*-Cr cells. Genes associated with T_CM_ (*Ccr7* and *Sell*) showed the opposite pattern of expression in *Klf2*- and *Klf3*-Cr cells. However, deficiency of either *Klf2* or *Klf3* resulted in reduced expression of genes that define LLEC (*Zeb2*, *Cx3cr1*, *S1pr5*) (Fig. 6*B*).

To investigate this further, we conducted GSEA against genes that are differentially expressed in T_CM_ vs. LLEC (55), for both *Klf2*- and *Klf3*-Cr memory CD8^+^ T cells (*SI Appendix*, Fig. S6 *B* and *C*). Notably, gene expression by *Klf2*- and *Klf3*-Cr cells both showed negative enrichment for the LLEC signature, suggesting both factors are required for production of LLEC. In contrast, *Klf3*-Cr cells showed a positive enrichment for the signature of T_CM_. This helps resolve the basis for the poor association of *Klf3*-Cr cells with the bulk recirculating signature (Fig. 5*D*), since that includes both T_CM_- and LLEC-associated genes. Unexpectedly, *Klf2*-Cr cells also showed some enrichment for the T_CM_ gene expression signature, albeit modest and not statistically significant.

The results of all GSEAs conducted on *Klf2*- and *Klf3*-Cr cells are summarized in Fig. 6*C* (and Dataset S7), providing an overview of the concordant and discordant effects of deleting these factors. When comparing the effects of *Klf2*-Cr and *Klf3*-Cr, we observe that some subsets of memory CD8^+^ T cells were differentially impacted (such as T_RM_ and T_CM_), indicating that KLF2 and KLF3 are in opposition, while for another subset (LLEC), loss of KLF2 and of KLF3 had similar effects, suggesting cooperativity. Ablation of neither *Klf2* nor *Klf3* led to an increase in the gene expression signature of exhausted T cells (Tr-T_EX_) (39) (Fig. 6*C*).

## Discussion

A notable feature of *Klf2* ablation was to induce rapid generation of T_RM_-like cells in the spleen and LNs. While some T_RM_ are generated in SLOs normally (9), the appearance of cells with these transcriptional traits as early as day 7 of the LCMV response implies that KLF2 loss directs precocious generation of T_RM_ at a stage where wild-type CD8^+^ T cells still exhibit effector cell characteristics. It is important to note that KLF2 expression is lost following TCR activation, but is normally restored by day 5 of the LCMV Armstrong response (17, 44), coinciding with migration of effector cells from lymphoid tissues into the blood and lymph.

Since our data clearly showed that KLF2 is required for access of effector and memory CD8^+^ T cells to the blood, KLF2 deficiency would be predicted to trap cells in the sites of priming (i.e., secondary lymphoid organs). Impaired access to the blood also likely explains why KLF2-deficient CD8^+^ T cells are not enriched in some NLTs (such as kidney and SI-IEL): Indeed, taking the paucity of *Klf2*-Cr cells in the blood into account, the frequency of KLF2-deficient cells in SI-IEL and kidney is elevated, relative to the control population, implying a competitive advantage for KLF2-deficient cells to form T_RM_ in NLT. This is further magnified in the liver, where there was a marked enrichment of KLF2-deficient T_RM_ despite poor blood access by those cells.

While our data are consistent with KLF2 loss leading to T_RM_-like differentiation and subsequent defective trafficking into the blood, it is possible that defective blood trafficking per se drives generation of T_RM_. This is an active area of investigation, although we unaware of studies that report changes in T_RM_ frequency following *S1pr1* gene ablation or exposure to S1PR inhibitors such as FTY720: Indeed, one of the few studies to examine this reported relatively normal induction of T_RM_ during FTY720 treatment of LCMV Armstrong–infected mice (56).

Our data indicate a complex relationship between KLF2 and KLF3. *Klf2* deficiency caused reduced *Klf3* transcription suggesting that KLF2 normally enhances KLF3 expression, yet *Klf2*- and *Klf3*- CRISPR led to opposing effects on T_RM_ and T_CM_ differentiation in the SLOs. Studies in erythrocyte differentiation indicated an incoherent feedback circuit for KLF3 and KLF1 (which is a close relative of KLF2), such that KLF1 induces expression of KLF3, but that KLF1 and KLF3 then induce and repress, respectively, the same target genes (51). KLF3 may also negatively regulate its own transcription, as indicated by reduced endogenous KLF3 expression in KLF3-transgenic B cells (23): likewise, we observed increased *Klf3* transcription when the *Klf3* coding sequence was disrupted by CRISPR. Perhaps reflecting the intricate interplay between these factors, ablation of *Klf2* and *Klf3* resulted in both concordant and discordant effects on memory CD8^+^ T cell generation. While loss of KLF2 provoked robust induction of T_RM_ in SLOs, *Klf3* deficiency resulted in a reduction in the SLO-T_RM_ pool. Nevertheless, it is important to note that *Klf3* deletion was not sufficient to substantially influence the generation of T_RM_ in NLTs. This implies KLF3 expression optimizes T_RM_ generation, at least in SLOs, but may be subservient to other factors, including a dominant effect of KLF2 loss, which is a signature feature of most CD8^+^ T cell T_RM_ populations reported (3, 5, 8). On the other hand, loss of either KLF2 or KLF3 led to a decline in the representation of cells expressing CX3CR1 and reduced transcription of *Cx3cr1*, *Zeb2,* and *S1pr5*, all of which are characteristic of both short- and long-lived effector CD8^+^ T cells (40, 55, 57, 58). This supports a model in which KLF2 and KLF3 oppose each other in terms of T_RM_ and T_CM_ differentiation, while they cooperate (or at least are both required) for generation of LLEC. GSEA and gene expression correlation analysis supports this interpretation.

This conclusion is analogous to the reported roles of KLF2 and KLF3 in B cell subset differentiation. We and others reported that KLF2-loss led to enhanced generation of MZ B cells, while Kirberg’s group showed that KLF3 deficiency reduced MZ-B frequencies, and that forced expression of KLF3 induced excess MZ-B generation (23, 25, 53, 59). Yet, deficiency in either KLF2 or KLF3 led to reduced frequencies of peritoneal B1 B cells (53, 54), suggesting positive roles for both factors. There is evidence that transcription of *Itgb7* (encoding β7-integrin) is induced by KLF2 and repressed by KLF3 in B cells, potentially involving competition for KLF binding sites (23, 25, 53, 59). Although β7-integrin expression is impaired in KLF2-deficient mature thymocytes (26), we observed no significant changes in *Itgb7* transcription in memory P14 CD8^+^ T cells subjected to *Klf2*- or *Klf3*-Cr (*SI Appendix*, Tables S1 and S5), suggesting that KLF-mediated regulation of *Itgb7* may change with T cell differentiation state. While specific targets of regulation may differ, our data support the model that KLF2 and KLF3 can act in opposition or in concert to regulate both B and T lymphocyte differentiation and trafficking.

Nevertheless, it is important to recognize that the basis for concordant and discordant effects of KLF2 and KLF3 deficiency in CD8^+^ T cells remains unclear. Both factors bind to similar DNA motifs, so it is possible some effects reflect competition for DNA binding, but the finding that KLF2 loss also leads to reduced KLF3 expression complicates interpretation, since it raises the possibility of epistatic interactions. Further studies will be needed to resolve the foundation for cooperative and antagonistic effects of KLF2 and KLF3 expression.

Recent studies exploring the response to chronic infections and tumors have proposed that KLF2 is required to support generation of effector cells and shield against exhaustion (20, 21). Furthermore, a recent report by Fagerberg et al. proposed that *Klf2*-deletion in P14 CD8^+^ T cells responding to acute LCMV infection drove phenotypic and functional characteristics of exhaustion (22). Those authors did not compare the gene expression characteristics of their KLF2-deficient population against T_RM_ signature genes, however, and it has been reported that several “exhaustion-related” genes are also expressed by T_RM_ produced in response to acute infection. Recent studies confirmed this but also identified gene expression differences that resolve CD8^+^ T cell T_RM_ from Tr-T_EX_ induced by acute and chronic antigen exposure, respectively (39). GSEA using these gene sets showed a positive correlation between *Klf2*-Cr memory CD8^+^ T cells and the T_RM_ signature (while *Klf3*-Cr memory CD8^+^ T cells had a negative correlation), while neither *Klf2*- or *Klf3*-Cr population correlated significantly with the Tr-T_EX_ gene expression signature. More importantly, our functional data are inconsistent with exhaustion or any other form of dysfunction in KLF2-deficient memory CD8^+^ T cells. We observed that KLF2-deficient memory cells behaved like control cells in terms of in vitro cytokine production and proliferation following TCR-activation, and in terms of expansion and protective immunity following in vivo recall stimulation. Notably, while we assessed the recall response of KLF2-deficient cells in situ (via heterologous infection), Fagerberg et al. utilized isolation and coadoptive transfer of *Klf2*-deficient and control memory CD8^+^ T cells prior to recall stimulation. KLF2-deficient cells show markedly elevated expression of ARTC2.2 (encoded by *Art2b*) and P2RX7, both of which can drive T cell death during isolation procedures (60, 61), and while Fagerberg et al. blocked ARTC2.2 they did not inhibit P2RX7 during isolation. Aside from these molecules, it is unclear whether KLF2-deficient cells, with their characteristics of T_RM_, efficiently reseed the spleen following adoptive transfer into the blood. This was not assessed by Fagerberg et al., complicating interpretation of their findings. Regardless, our findings indicate that KLF2-deficient memory CD8^+^ T cells are at least as competent as control cells in cytokine production, expansion, and pathogen control, three of the core functions of adaptive memory. A distinct question is whether KLF2-deficient memory cells have similar functional characteristics as normal T_RM_ from the same organs. Future studies investigating this issue will be important to resolve whether, in addition to phenotypic and transcriptional similarities to T_RM_, and a similar inability to recirculate, KLF2 deficiency promotes adoption of T_RM_ functional traits, that may vary by tissue location (62).

Overall, our studies indicate that KLF2 expression directs effector T cells into becoming T_CIRCM_ and overrides the differentiation of T_RM_, which appears to occur by default when KLF2 is defective. KLF3 acts in concert with KLF2 for generation of some T_CIRCM_ subsets (especially LLEC), but the two factors have reciprocal effects on generation of T_RM_ and T_CM_. Taken together, these data indicate that KLFs play a central role in driving the production of resident vs. recirculating memory CD8^+^ T cells.

## Materials and Methods

### Mice.

C57BL/6 (B6: CD45.2) and C57BL/6J-Ptprcem6Lutzy/J (JAXBoy: CD45.1) mice were purchased from The Jackson Laboratory. KLF2-GFP mice have been previously described (27) and were bred to LCMV-GP33/D^b^-specific TCR transgenic P14 mice and intercrossed with JAXBoy mice to generate congenically distinct offspring. Animals were maintained under specific-pathogen-free conditions at the University of Minnesota. In all experiments, mice were randomly assigned to experimental groups. All experimental procedures were approved by the Institutional Animal Care and Use Committee at the University of Minnesota.

### CRISPR/Cas9 Nucleofection and Adoptive Transfer.

Congenically distinct KLF2-GFP P14 CD8+ T cells were activated with plate-bound αCD3 and αCD28 and rhIL-2 for 48 h, incubated with sgRNAs/Cas9 complexes for target (*Klf2* or *Klf3*) or control (*Cd19* or *Thy1*) genes, and electroporated with a Lonza 4DNucleofector X Unit, using published conditions (63). Following culture in IL-2 for 24 to 48 h, 5 × 10^4^ CRISPR-modified cells were coadoptively transferred into congenically distinct host mice, which received LCMV-Arm infection (2 × 10^5^ PFU) within 2 h of transfer.

Detailed experimental procedures are provided in *SI Appendix*.

### Isolation of Lymphocytes from Tissues.

Mice were intravenously injected with ARTC2.2-blocking nanobody S+16a (Treg-protector, BioLegend) at least 15 min prior to organ harvest as described (46, 47). To label vascular-associated circulating lymphocytes in NLT, i.v. injection of PerCP-Cy5.5-conjugated CD8α antibody was performed as previously described (17, 64). Lymphocytes were isolated from tissues including the spleen, inguinal lymph nodes, small intestine epithelium (SI-IEL), and kidney and peripheral blood as previously described (17).

### Flow Cytometry.

Direct ex vivo staining was performed after lymphocyte isolation as previously described (17, 45). Flow cytometric analysis was performed on an LSRFortessa (BD Biosciences) or Cytek Aurora, and data were analyzed using FlowJo software (BD Biosciences).

### RNA-seq and Bioinformatic Analysis.

CRISPR-modified donor P14 CD8^+^ T cells were FACS sorted from the spleen, 8- or 25-d post–LCMV infection, RNA isolated, and submitted for sequencing through the University of Minnesota Genomics Center, as previously described (55). FastQC v0.12.1 (65) was used to generate sequence quality reports for raw and trimmed reads. featureCounts v2.0.6 (66) was used to count mapped reads to genes. *Mus musculus* GRC build 39.109 gtf file was used as the reference file for gene count mapping.

Detailed experimental procedures are provided in *SI Appendix*.

### CUT&Tag and Bioinformatic Analysis.

Naïve and activated CD8^+^ T cells (the latter from d8 LCMV Armstrong–infected mice) were enriched. Cell nuclei were isolated, bound to Concanavalin A-beads, then incubated with anti-KLF2 antibody (or control Rabbit IgG) overnight. After washing, a secondary anti-rabbit was followed with CUTANA™ pAG-Tn5. Tagmentation was performed in a thermocycler. Following DNA isolation, libraries were amplified with Singular Genomics sequencing adaptors, and paired-end sequencing (50 cycles) was performed on a Singular Genomics G4 sequencer, generating ~15 million reads per sample. Raw sequencing data were processed using the nf-core/cutandrun pipeline (v3.2.2) (https://nf-co.re/cutandrun/3.2.2). Peaks were annotated using HOMER (v4.9.1), and heatmaps were generated with deepTools (v3.3.0).

Detailed experimental procedures are provided in *SI Appendix*.

### Statistical Analysis.

Student’s *t* test (paired or unpaired, as indicated in figure legends) was used when comparing two groups, while comparisons between more than two groups used one-way ANOVA. All analysis used GraphPad 10.0 Software. Averages are indicated as medians, with error bars representing SEM. Symbols indicate *P*-values of <0.05 (*), <0.01 (**), <0.001 (***), or <0.0001 (****).

Additional details on statistical methods are provided in *SI Appendix*.

RNAseq and CUT&Tag data are deposited at GEO with the following Accession Numbers: (TBD—in process).

## Supplementary Material

Appendix 01 (PDF)

Dataset S01 (XLSX)

Dataset S02 (XLSX)

Dataset S03 (XLSX)

Dataset S04 (XLSX)

Dataset S05 (XLSX)

Dataset S06 (XLSX)

Dataset S07 (XLSX)

## Data Availability

Genomics analysis data have been deposited in GEO (GSE324376 and GSE322657) (67, 68).
