# Supplementary material for: KLF2 overrides the resident memory CD8 T cell differentiation program, in opposition to KLF3
Source: Proc Natl Acad Sci U S A. 2026 Mar 23;123(13):e2533700123. doi: 10.1073/pnas.2533700123 (PMC13037849; doi:10.1073/pnas.2533700123)
Supplement: Supplementary file 1 — Appendix 01 (PDF) [file pnas.2533700123.sapp.pdf]

SI Appendix

KLF2 overrides the resident memory CD8 T cell differentiation program, in opposition to KLF3

Taylor A. DePauw[1], Kexin Gai[3], Jian Shen[3], Nicholas J. Maurice[1], Ka Hyun Rhee[1], William J. Valente[1] Christine H. O'Connor[2], Weiguo Cui[3], Changwei Peng[1]*, Stephen C. Jameson[1]*

<u>Inventory of Supporting Material</u>

Supplementary Materials and Methods
Supplementary References
Figures S1-S6, preceded by figure legends
Descriptions of Datasets S1-S7

Supplemental Materials and Methods

CRISPR/Cas9 RNP nucleofection

On the day of nucleofection, RNP complex at 3:1 ratio of sgRNA with Cas9 (150pmol sgRNA : 50pmol Cas9l) was assembled and incubated at RT for 10 minutes. 1 to 10 million CD8+ T cells were resuspended in 20uL of P4 primary cell nucleofection solution (Lonza) and further incubated with Cas9 RNP for 2min at RT. Then the cell-RNP mixture was nucleofected using a Lonza 4DNucleofector X Unit as previously described, using program CM137 (1-3).

Two sgRNAs were used for efficient deletion of each target gene:
sgKlf2_1: 5'-CUGGCCGCGAAAUGAACCCG-3'
sgKlf2_2: 5'-UCCAUGGGAUUGGACGGUCU-3'
sgKlf3_1: 5'-CCUCACUCACGGGAUACAGG -3'
sgKlf3_2 5'-GGCGAGUACUUCUUAAUGGG -3'
sgCd19_1: 5'-CCUGGCCUGGGAUUGCACGU-3'
sgCd19_2: 5'-GAGAAGCUGGCUUGGUAUCG-3'
sgThy1_1: 5'-CAGUCUUGCAGGUGUCCCGA -3'
sgThy1_2: 5'-CCGCCAUGAGAAUAACACCA -3'

ICE Analysis

ICE analysis was conducted on P14 CD8$^+$ T cells subjected to CRISPR/Cas9 nucleofection with sgRNAs targeting *Klf2*, *Klf3* or *Thy1*. Both in vitro cultured cells (1x10^6 cells, isolated at least 24 hours post nucleofection) and cells sorted ex vivo after adoptive transfer and LCMV infection (at least 2.5x10^5 cells isolated 20 days post infection) were assayed. Genomic DNA was isolated using the DNeasy Blood & Tissue Kit (Qiagen) and loci of interest amplified from purified DNA using NEBNext High-Fidelity 2× PCR Master Mix (New England Biolabs) according to the manufacturer's instructions. The following primer pairs were used for PCR and sequencing:

sgKlf3_1_Fw: 5′-CCATCAGCCTTCTCACTGAAT-3′;
sgKlf3_1_Rv: 5′-GGTGTCCTCACCAGACTACTA-3′;
sgKlf2_1_Fw: 5′-GCCTCAGGGAGTTAGACTTCA-3′;
sgKlf2_2_Rv: 5′-GGGCTCGGCCTTCACTA-3′.

PCR amplicons were resolved on a 1% agarose gel, and bands of the expected size were excised and purified using the Zymoclean Gel DNA Recovery Kit (Zymo Research). Purified products were Sanger-sequenced (ACGT, Inc.), and resulting chromatograms analyzed using the ICE (Inference of CRISPR Edits) webtool from Synthego. Examples of ICE analysis from sorted cells are provided in Supplemental Figures 1B and 5A. These were consistently >90% for Klf2 and ranged from ~50%->95% for Klf3.

Details of RNA-seq and bioinformatic analysis

CD8$^+$ T cells were isolated from the spleen, 8- or 25-days post LCMV infection and FACS sorted (Aria II, BD Bioscience), based on CD8α, CD45.2 and CD45.1 expression. RNA was isolated using QIAGEN RNeasy Plus Mini Kit and submitted for library generation and sequencing (HiSeq 2500, paired end read, 125 bp). Bioinformatic analysis was done in R (v 4.4.3) (https://www.R-project.org/). Bulk RNAseq samples were processed and aligned using the CHURP version 1.0.0 command line interface framework (4). A full description of the CHURP pipeline can be found in Baller et al., 2019 (4). Raw count data were first pre-filtered to remove lowly expressed genes, retaining only those with at least 10 counts across a minimum of three samples. Subsequent normalization and differential expression analyses were conducted using

the DESeq2 package (v 1.44.0) (5). Size-normalized counts were used to visualize gene expression levels across groups, while variance-stabilizing transformation (VST)-normalized counts were used for heatmap generation. For heatmap visualization, row Z-scores were further calculated to facilitate comparison across genes. Differential expression analysis was performed between KLFs-Cr and their corresponding Ctrl-Cr samples within each time point (D7 or D25). Differentially expressed genes (DEGs) were identified from DESeq2 results after log fold change shrinkage (lfcShrink) using the apeglm method. DEGs were classified by: padj < 0.05 and Log2FC > 1 as “High” significance; padj < 0.05 and 0.58 ≤ Log2FC ≤ 1 as “Moderate” significance. Gene set enrichment analysis (GSEA) was performed using the ClusterProfler (v 4.12.6) (6). Custom gene sets from Milner et al. (7), Scott et al. (8), Park et al. (9) and Renkema et al. (10) were used for enrichment analysis. Genes were ranked based on differential expression results using the metric sign(log2FoldChange) × −log10(adjusted P value). Enrichment analysis was conducted with the GSEA function in clusterProfiler, using 1,000 permutations and Benjamini–Hochberg (BH) correction for multiple testing. Results were visualized using dot plots displaying gene ratio and BH-adjusted P values.

RNAseq data are available at GEO, Accession Number: GSE324376 (11).

Details of CUT&Tag assay and bioinformatic analysis
Naïve CD8 T cells were isolated from spleens using the EasySep™ Mouse Naïve CD8+ T Cell Isolation Kit (Stemcell, #19858). Total CD8 T cells were isolated from spleens and lymph nodes of LCMV Armstrong-infected mice at day 8 post-infection using the EasySep™ Mouse CD8+ T Cell Isolation Kit (Stemcell, #19853). Cells from different mice were used as biological replicates. Sequencing libraries were prepared as follows, based largely on the Bench Top CUT&Tag v3 protocol (dx.doi.org/10.17504/protocols.io.bcuhiwt6): 200,000 live, unfixed cells per sample were lysed to extract nuclei, which were then bound to concanavalin A-coated beads (EpiCypher, #21-1401). Bead-bound nuclei were incubated overnight at 4°C with 1 µL of anti-KLF2 antibody (Millipore Sigma, #09-820) or 0.5 µL of Rabbit IgG Negative Control Antibody (EpiCypher, #13-0042) on a nutator. The following day, nuclei were washed and incubated for 30 min at room temperature (RT) with a 1:100 dilution of Anti-Rabbit Secondary Antibody (EpiCypher, #13-0047), followed by a 1-hour incubation with CUTANA™ pAG-Tn5 (EpiCypher, #15-1117). Tagmentation was performed in 50 µL of Tagmentation Buffer for 1 hour at 37°C in a thermocycler. The reaction was stopped by adding 3 µL of 0.5M EDTA, 1 µL of 10% SDS, and 1 µL of Proteinase K (Sigma, #3115879001, 25 mg/10 mL in H_2O), followed by incubation at 50°C for 1 hour. DNA was extracted using Phenol:Chloroform:Isoamyl Alcohol (25:24:1, v/v) (Thermo Fisher, #15593031) and purified with the MinElute Reaction Cleanup Kit (Qiagen, #28204). Libraries were amplified with Singular Genomics sequencing adaptors and paired-end sequencing (50 cycles) was performed on a Singular Genomics G4 sequencer, generating ~15 million reads per sample.

Raw sequencing data were processed using the nf-core/cutandrun pipeline (v3.2.2) (https://nf-co.re/cutandrun/3.2.2). Quality control of the libraries was assessed using FastQC (v0.11.8) (12). Reads were aligned to the M. musculus mm10 genome using Bowtie2 (v2.2.5). Peak calling was performed with MACS2 using default pipeline settings. Peaks were annotated using HOMER (v4.9.1), and heatmaps were generated with deepTools (v3.3.0). Representative genome tracks were visualized using the WashU Epigenome Browser.

KLF2 CUT&Tag data are available at GEO, Accession Number: GSE322657 (13).

Statistical analysis

Paired or unpaired two-tailed Student's t-test (for parametric test) was used when comparing two groups, as described in the figure legends. For comparisons with more than two groups, statistical differences were calculated using Ordinary one-way ANOVA involving Tukey's multiple comparisons test. Data in Fig. 1C, 1D, 3C and 5A were log transformed before statistical analysis. All experiments were analyzed using Prism 10 (GraphPad Software). Samples are shown with medians and error bars showing the SEM. Statistical significance is indicated in the figures as follows: $p>0.05$ ("ns" for not significant); $p<0.05$ (*); $p<0.01$ (**); $p<0.001$ (***); $p<0.0001$ (****).

Supplemental References

1. A. Seki, S. Rutz, Optimized RNP transfection for highly efficient CRISPR/Cas9-mediated gene knockout in primary T cells. *J Exp Med* **215**, 985-997 (2018).
2. H. Borges da Silva *et al.*, Sensing of ATP via the Purinergic Receptor P2RX7 Promotes CD8(+) Trm Cell Generation by Enhancing Their Sensitivity to the Cytokine TGF-beta. *Immunity* **53**, 158-171 e156 (2020).
3. D. A. Walsh *et al.*, The Functional Requirement for CD69 in Establishment of Resident Memory CD8(+) T Cells Varies with Tissue Location. *J Immunol* **203**, 946-955 (2019).
4. J. Baller, T. Kono, A. Herman, Y. Zhang, CHURP: A Lightweight CLI Framework to Enable Novice Users to Analyze Sequencing Datasets in Parallel.
5. M. I. Love, W. Huber, S. Anders, Moderated estimation of fold change and dispersion for RNA-seq data with DESeq2. *Genome Biol* **15**, 550 (2014).
6. G. Yu, L. G. Wang, Y. Han, Q. Y. He, clusterProfiler: an R package for comparing biological themes among gene clusters. *OMICS* **16**, 284-287 (2012).
7. J. J. Milner *et al.*, Runx3 programs CD8(+) T cell residency in non-lymphoid tissues and tumours. *Nature* **552**, 253-257 (2017).
8. M. C. Scott *et al.*, Deep profiling deconstructs features associated with memory CD8(+) T cell tissue residence. *Immunity* **58**, 162-181 e110 (2025).
9. S. L. Park *et al.*, Tissue-resident exhausted and memory CD8(+) T cells have distinct ontogeny, function and role in disease. *Nat Immunol* **27**, 110-125 (2026).
10. K. R. Renkema *et al.*, KLRG1(+) Memory CD8 T Cells Combine Properties of Short-Lived Effectors and Long-Lived Memory. *J Immunol* **205**, 1059-1069 (2020).
11. T. A. DePauw *et al.* (2026) RNAseq data (from "KLF2 overrides the resident memory CD8 T cell differentiation program, in opposition to KLF3"). (GEO Accession Number GSE324376). Deposited March 9th 2026. https://www.ncbi.nlm.nih.gov/geo/query/acc.cgi?&acc=GSE324376
12. S. Andrews, FastQC: a quality control tool for high throughput sequence data. (2010).
13. T. A. DePauw *et al.* (2026) KLF2 CUT&Tag data (from "KLF2 overrides the resident memory CD8 T cell differentiation program, in opposition to KLF3"). (GEO Accession Number GSE322657). Deposited March 2nd 2026. https://www.ncbi.nlm.nih.gov/geo/query/acc.cgi?&acc= GSE322657

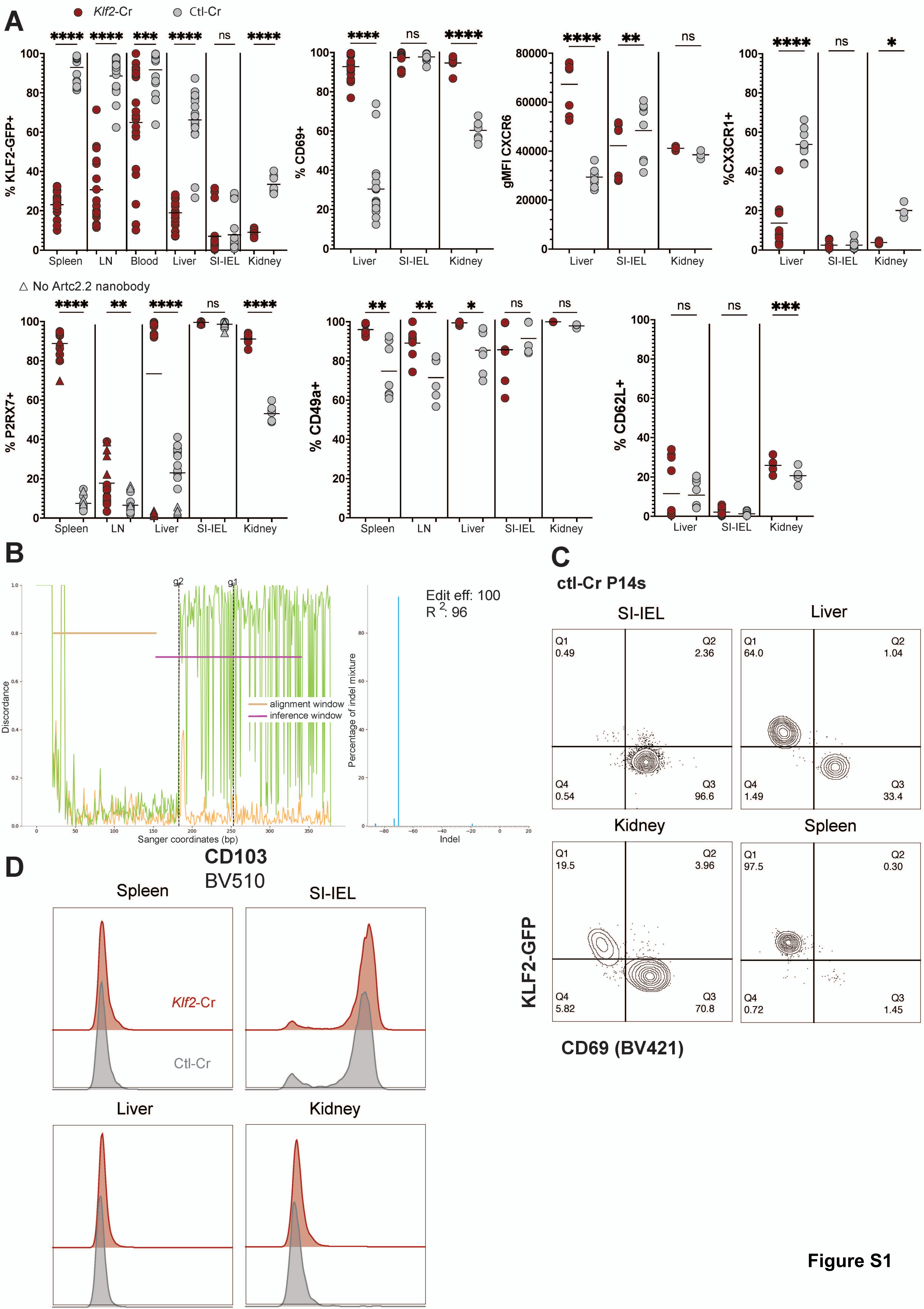
A
Klf2-Cr
Ctl-Cr
% KLF2-GFP+
Spleen
LN
Blood
Liver
SI-IEL
Kidney
% CD69+
gMFI CXCR6
%CX3CR1+
△ No Artc2.2 nanobody
% P2RX7+
% CD49a+
% CD62L+
****
***
**
*
ns
B
Edit eff: 100
R 2: 96
alignment window
inference window
Discordance
Sanger coordinates (bp)
Percentage of indel mixture
Indel
g2
g1
C
ctl-Cr P14s
SI-IEL
Liver
Kidney
Spleen
Q1 0.49
Q2 2.36
Q4 0.54
Q3 96.6
Q1 64.0
Q2 1.04
Q4 1.49
Q3 33.4
Q1 19.5
Q2 3.96
Q4 5.82
Q3 70.8
Q1 97.5
Q2 0.30
Q4 0.72
Q3 1.45
KLF2-GFP
CD69 (BV421)
D
CD103
BV510
Spleen
SI-IEL
Liver
Kidney
Klf2-Cr
Ctl-Cr

Figure S1

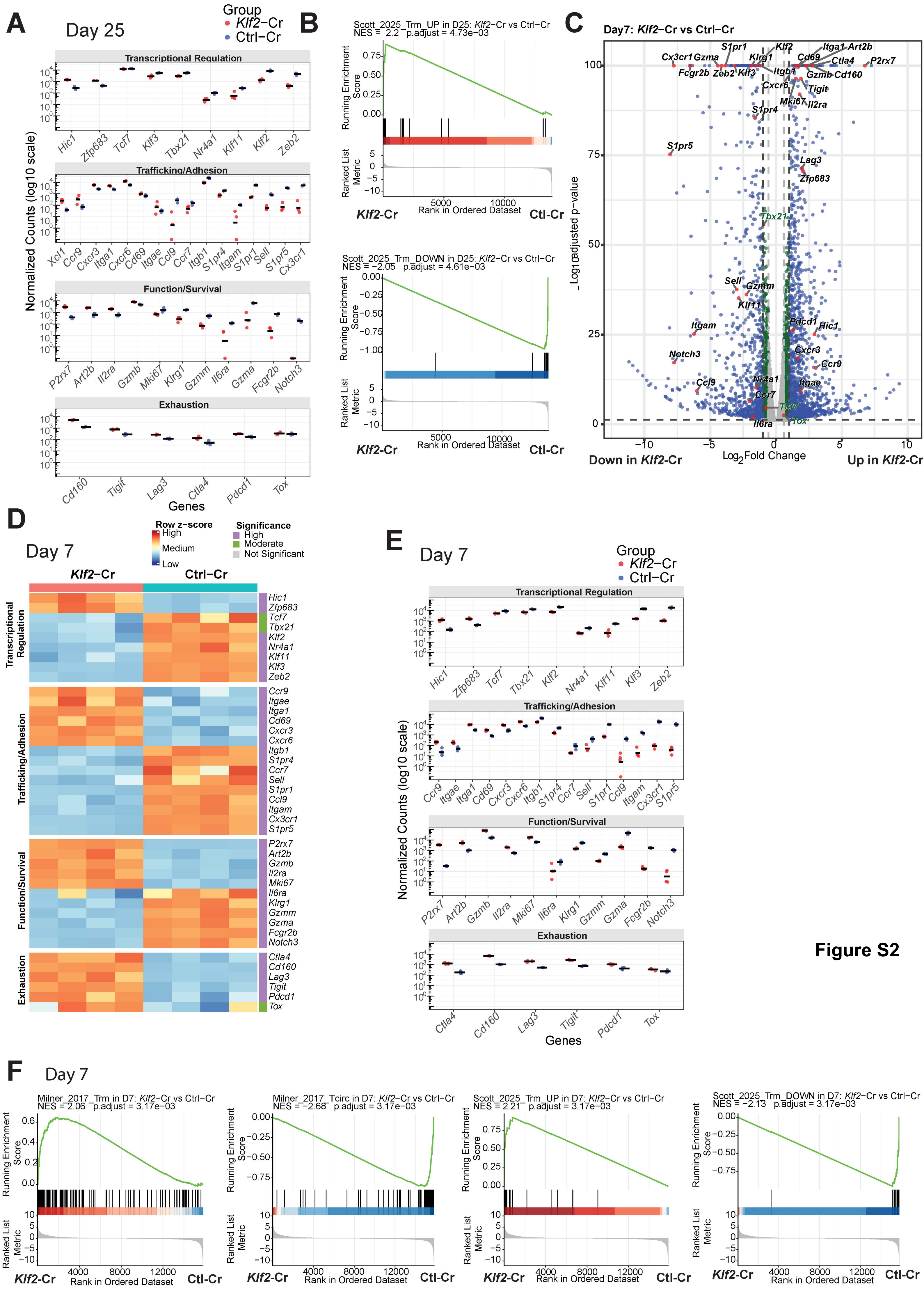
A
Day 25
Group
Klf2−Cr
Ctrl−Cr
Transcriptional Regulation
Trafficking/Adhesion
Function/Survival
Exhaustion
Normalized Counts (log10 scale)
Genes
B
Scott_2025_Trm_UP in D25: Klf2−Cr vs Ctrl−Cr
NES = 2.2 p.adjust = 4.73e−03
Scott_2025_Trm_DOWN in D25: Klf2−Cr vs Ctrl−Cr
NES = −2.05 p.adjust = 4.61e−03
Running Enrichment Score
Ranked List Metric
Rank in Ordered Dataset
Klf2-Cr
Ctl-Cr
C
Day7: Klf2−Cr vs Ctrl−Cr
−Log10 adjusted p−value
Log2Fold Change
Down in Klf2-Cr
Up in Klf2-Cr
D
Day 7
Row z-score
High
Medium
Low
Significance
High
Moderate
Not Significant
Klf2−Cr
Ctrl−Cr
Transcriptional Regulation
Trafficking/Adhesion
Function/Survival
Exhaustion
E
Day 7
Group
Klf2−Cr
Ctrl−Cr
Normalized Counts (log10 scale)
Genes
F
Day 7
Milner_2017_Trm in D7: Klf2−Cr vs Ctrl−Cr
NES = 2.06 p.adjust = 3.17e−03
Milner_2017_Tcirc in D7: Klf2−Cr vs Ctrl−Cr
NES = −2.68 p.adjust = 3.17e−03
Scott_2025_Trm_UP in D7: Klf2−Cr vs Ctrl−Cr
NES = 2.21 p.adjust = 3.17e−03
Scott_2025_Trm_DOWN in D7: Klf2−Cr vs Ctrl−Cr
NES = −2.13 p.adjust = 3.17e−03
Running Enrichment Score
Ranked List Metric
Rank in Ordered Dataset
Klf2-Cr
Ctl-Cr

Figure S2

A

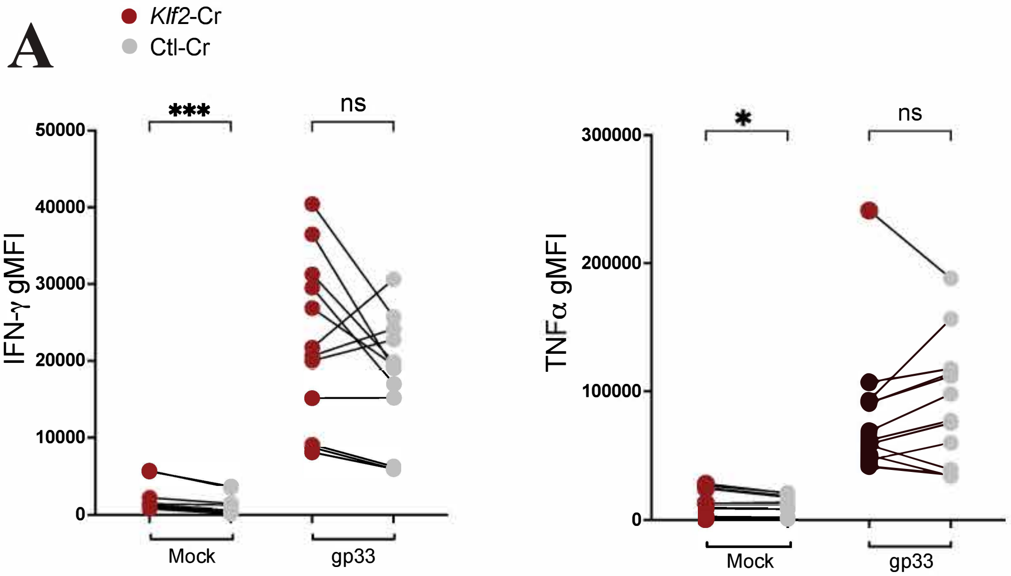
Klf2-Cr
Ctl-Cr
***
ns
IFN-γ gMFI
50000
40000
30000
20000
10000
0
Mock
gp33
*
ns
TNFα gMFI
300000
200000
100000
0
Mock
gp33

B

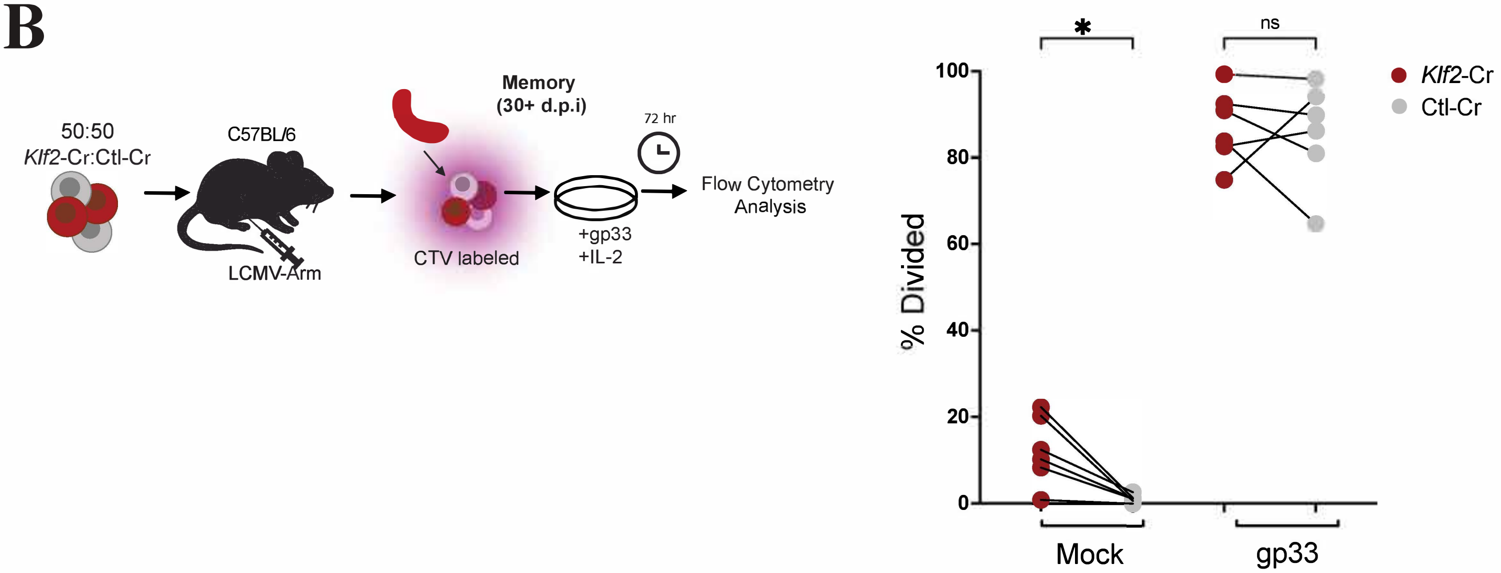
50:50
Klf2-Cr:Ctl-Cr
C57BL/6
LCMV-Arm
Memory
(30+ d.p.i)
CTV labeled
72 hr
+gp33
+IL-2
Flow Cytometry
Analysis
*
ns
Klf2-Cr
Ctl-Cr
% Divided
100
80
60
40
20
0
Mock
gp33

Figure S3

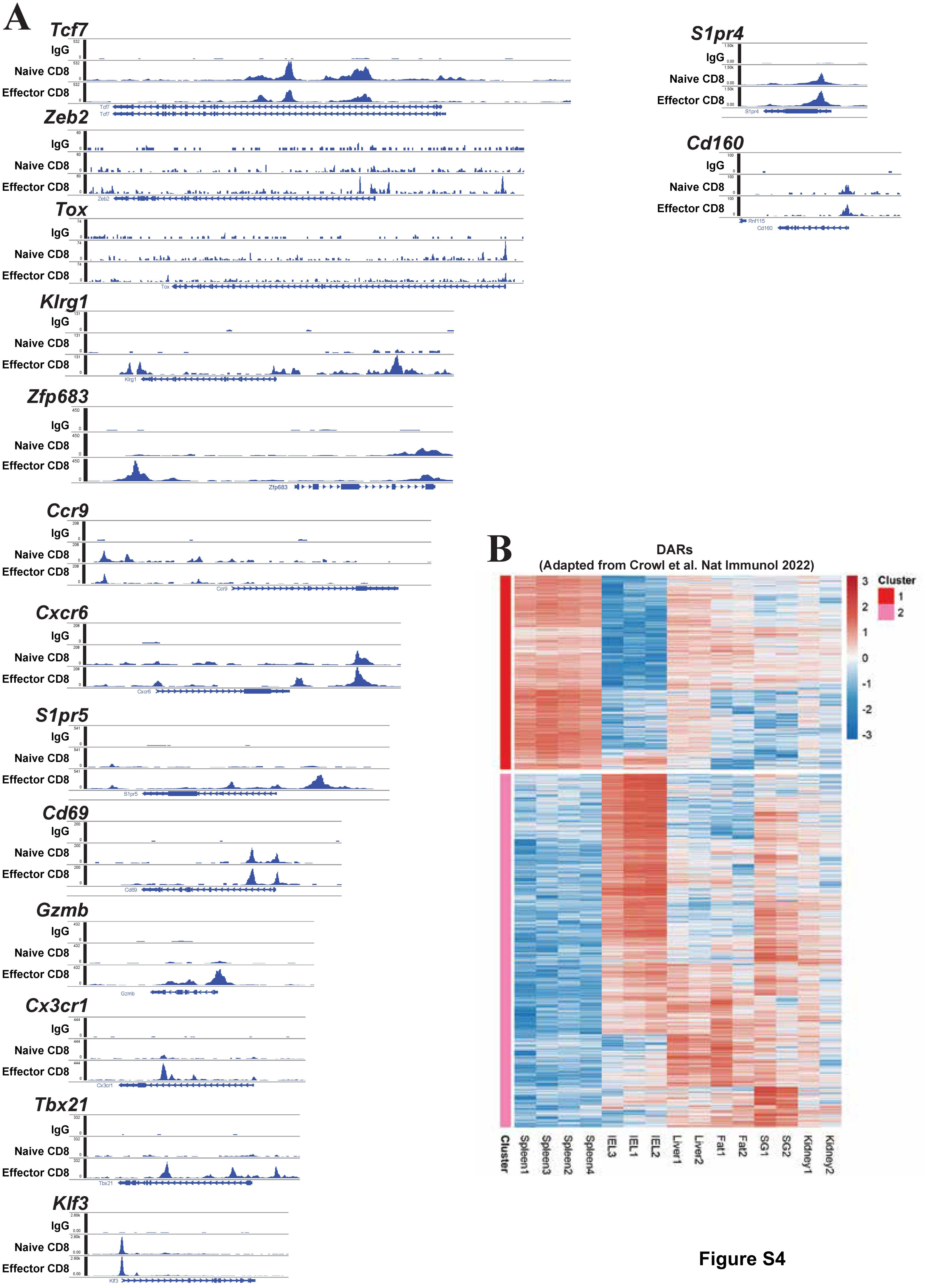

A
Tcf7
IgG
Naive CD8
Effector CD8
Zeb2
Tox
Klrg1
Zfp683
Ccr9
Cxcr6
S1pr5
Cd69
Gzmb
Cx3cr1
Tbx21
Klf3
S1pr4
Cd160
Rnf115
B
DARs
(Adapted from Crowl et al. Nat Immunol 2022)
Cluster
1
2
3
2
1
0
-1
-2
-3
Cluster
Spleen1
Spleen3
Spleen2
Spleen4
IEL3
IEL1
IEL2
Liver1
Liver2
Fat1
Fat2
SG1
SG2
Kidney1
Kidney2

Figure S4

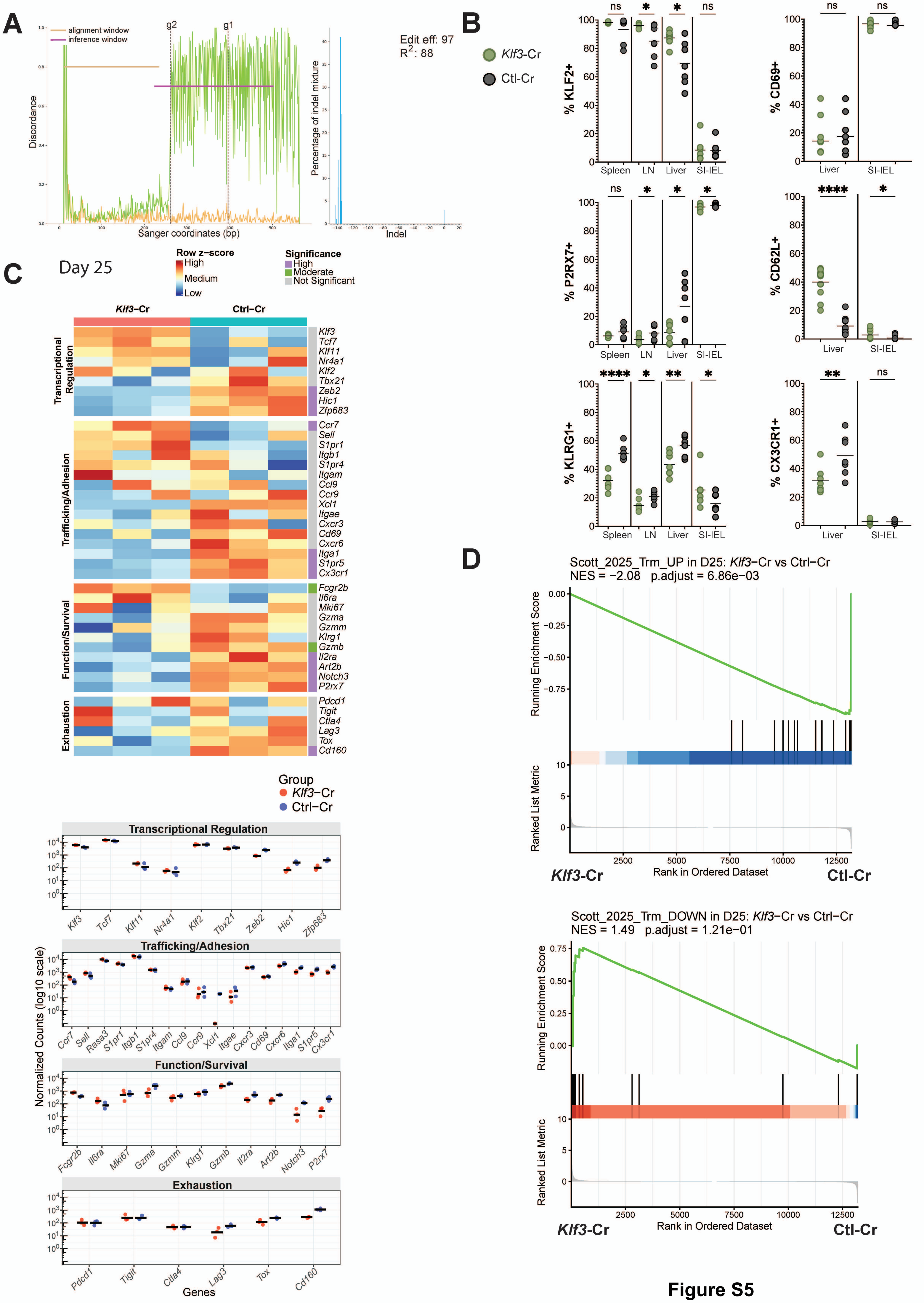
A
alignment window
inference window
g2
g1
Discordance
Sanger coordinates (bp)
Percentage of indel mixture
Indel
Edit eff: 97
R2: 88
B
Klf3-Cr
Ctl-Cr
% KLF2+
% CD69+
% P2RX7+
% CD62L+
% KLRG1+
% CX3CR1+
ns
*
**
****
Spleen
LN
Liver
SI-IEL
C
Day 25
Row z-score
High
Medium
Low
Significance
High
Moderate
Not Significant
Klf3-Cr
Ctrl-Cr
Transcriptional Regulation
Trafficking/Adhesion
Function/Survival
Exhaustion
Klf3
Tcf7
Klf11
Nr4a1
Klf2
Tbx21
Zeb2
Hic1
Zfp683
Ccr7
Sell
S1pr1
Itgb1
S1pr4
Itgam
Ccl9
Ccr9
Xcl1
Itgae
Cxcr3
Cd69
Cxcr6
Itga1
S1pr5
Cx3cr1
Fcgr2b
Il6ra
Mki67
Gzma
Gzmm
Klrg1
Gzmb
Il2ra
Art2b
Notch3
P2rx7
Pdcd1
Tigit
Ctla4
Lag3
Tox
Cd160
Group
Klf3-Cr
Ctrl-Cr
Rasa3
Normalized Counts (log10 scale)
Genes
D
Scott_2025_Trm_UP in D25: Klf3-Cr vs Ctrl-Cr
NES = −2.08 p.adjust = 6.86e−03
Running Enrichment Score
Ranked List Metric
Rank in Ordered Dataset
Klf3-Cr
Ctl-Cr
Scott_2025_Trm_DOWN in D25: Klf3-Cr vs Ctrl-Cr
NES = 1.49 p.adjust = 1.21e−01

Figure S5

A

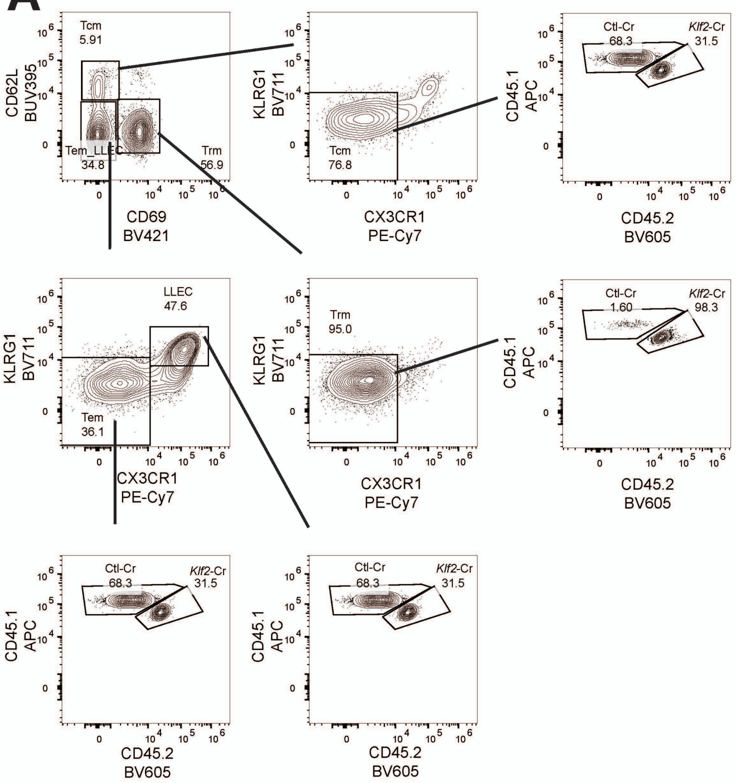

B

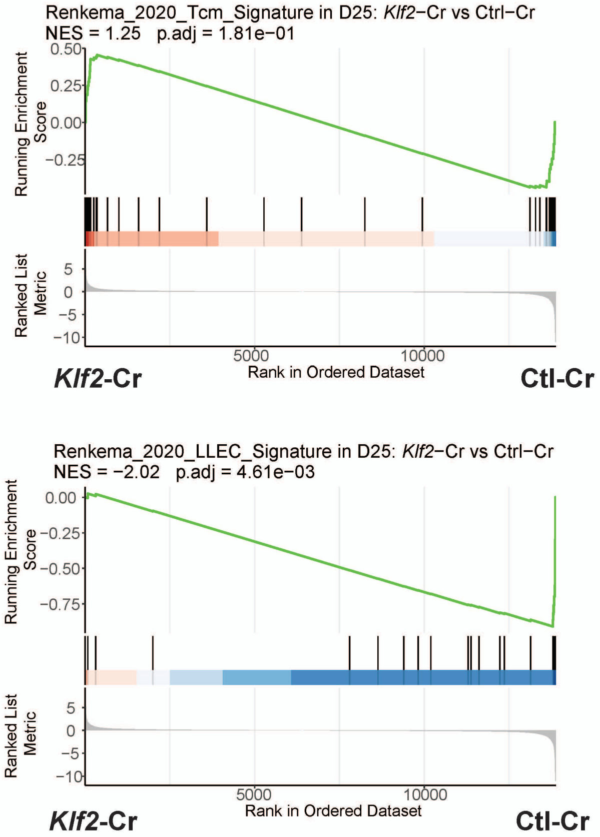

C

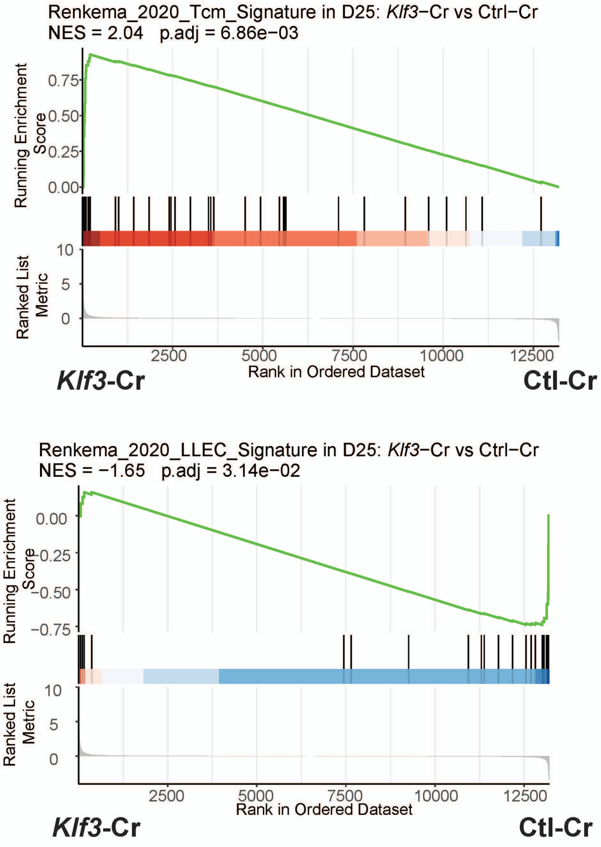

Figure S6

Supplemental Datasets:

Below is a description of the contents of the 7 supplemental Datasets submitted with **DePauw et al.**

Dataset S1: RNAseq of *Klf2*-Cr and control (*Cd19*-Cr) P14 $CD8^+$ T cells, isolated from the spleens of three mice, 25 days after LCMV infection. The data are sorted based on statistical significance and average fold change.

Dataset S2: RNAseq of *Klf2*-Cr and control (*Cd19*-Cr) P14 $CD8^+$ T cells, isolated from the spleens of three mice, 7 days after LCMV infection. The data are sorted based on statistical significance and average fold change.

Dataset S3: KLF2 CUT&Tag data on naïve and effector $CD8^+$ T cells (isolated at day 8 following LCMV infection), from C57BL/6 mice. Representative KLF2 occupancy peaks are identified by genomic location and nearest documented gene.

Dataset S4: Reanalysis of ATAC-seq clusters reported by Crowl et al. (ref 41). Clusters 1-3 of Crowl et al. were combined to generate “Cluster 1”, while Clusters 4-8 of Crowl et al. were combined into “Cluster 2”.

Dataset S5: Alignment of KLF2 CUT&Tag data with ATAC-seq data from Crowl et al. (ref 41). The tabs show the KLF2 occupancy (from representative data of an effector $CD8^+$ T cell sample) in the two DAR clusters described in Dataset S4.

Dataset S6: RNAseq of *Klf3*-Cr and control (*Cd19*-Cr) P14 $CD8^+$ T cells, isolated from the spleens of three mice, 25 days after LCMV infection. The data are sorted based on statistical significance and average fold change.

Dataset S7: Details of GSEA analysis using gene sets from Klf2-Cr vs Cd19-Cr P14 T cells (d25 and d7 post-LCMV) and Klf3-Cr vs Cd19-Cr P14 T cells (day 25 post-LCMV). Core enrichment genes are listed.
